# Genome Evolution in the *Eremothecium* Clade of the *Saccharomyces* Complex Revealed by Comparative Genomics

**DOI:** 10.1534/g3.111.001032

**Published:** 2011-12-01

**Authors:** Jürgen Wendland, Andrea Walther

**Affiliations:** Carlsberg Laboratory, Yeast Biology, Valby 2500, Denmark

**Keywords:** whole-genome sequencing, genome evolution, ancestral gene order, GC content, mating type locus

## Abstract

We used comparative genomics to elucidate the genome evolution within the pre–whole-genome duplication genus *Eremothecium*. To this end, we sequenced and assembled the complete genome of *Eremothecium cymbalariae*, a filamentous ascomycete representing the *Eremothecium* type strain. Genome annotation indicated 4712 gene models and 143 tRNAs. We compared the *E. cymbalariae* genome with that of its relative, the riboflavin overproducer *Ashbya (Eremothecium) gossypii*, and the reconstructed yeast ancestor. Decisive changes in the *Eremothecium* lineage leading to the evolution of the *A. gossypii* genome include the reduction from eight to seven chromosomes, the downsizing of the genome by removal of 10% or 900 kb of DNA, mostly in intergenic regions, the loss of a TY3-Gypsy–type transposable element, the re-arrangement of mating-type loci, and a massive increase of its GC content. Key species-specific events are the loss of *MNN1*-family of mannosyltransferases required to add the terminal fourth and fifth α-1,3-linked mannose residue to *O*-linked glycans and genes of the *Ehrlich* pathway in *E. cymbalariae* and the loss of ZMM-family of meiosis-specific proteins and acquisition of riboflavin overproduction in *A. gossypii*. This reveals that within the *Saccharomyces* complex genome, evolution is not only based on genome duplication with subsequent gene deletions and chromosomal rearrangements but also on fungi associated with specific environments (*e.g.* involving fungal-insect interactions as in *Eremothecium*), which have encountered challenges that may be reflected both in genome streamlining and their biosynthetic potential.

Studying yeast genomes provides crucial insight into the evolution of these genomes on the molecular level. One large class known as hemiascomycetes contains the budding yeasts with *Saccharomyces cerevisiae*, which was the first eukaryotic species whose genome was sequenced (Goffeau 1996). This endeavor has been followed by sequencing efforts using next-generation sequencing methodology, which provided a wealth of information, particularly for species in the *Saccharomyces* complex (Dujon *et al.*, 2004; Cuomo and Birren 2010).

Compilation of large amounts of sequence data can be used to generate a compendium of genes shared by a common ancestor prior to the breakup of lineages. Ideally, such an ancestral genome can be established for any well-established node in a phylogenetic tree. One such effort using 11 yeast species provided a reconstructed genome of an ancestor of the yeast lineage just prior to the whole-genome duplication (Gordon *et al.*, 2009).

With reference to such an ancestor, one can infer events that must have occurred in the respective lineages that led to the current species’ genomes. This may provide significant insight in understanding the biology of these species in terms of adaptive evolution and the acquisition of species-specific traits. Given the relative ease in molecular genetic manipulation of most fungi in the *Saccharomyces* complex, this wealth of novel information may direct our roads of research to study the functional relevance of alterations detected by comparative genomics (Rusche and Rine 2010).

The genus *Eremothecium* has been assigned to clade 12 of the *Saccharomyces* complex (Kurtzman and Robnett 2003). With *Ashbya gossypii*, this genus contains an industrially important species for the production of riboflavin (vitamin B_2_). The molecular biology involved in riboflavin production has been studied for many years in *A. gossypii*. This led to the identification of pathways required for providing GTP as a central precursor for riboflavin biogenesis (Förster *et al.*, 1999; Jimenez *et al.*, 2005; Mateos *et al.*, 2006).

In addition, interest in Ashbya was spurred by the unusual filamentous growth mode within the *Saccharomyces* complex (Wendland and Philippsen 2001). Current hypotheses favor the notion that an *Eremothecium* ancestor has gained the potential to generate true hyphae instead of employing pseudohyphal growth (Schmitz and Philippsen 2011). Therefore, studying the *Eremothecium* genus in more detail may provide us with molecular insight on which steps may have been involved to establish filamentous growth in this genus.

*E. cymbalariae*, *Eremothecium ashbyi*, and *A. gossypii* are filamentous fungi that share a rare growth mode that includes dichotomous tip branching. *Eremothecium ashbyi* and *A. gossypii* are both flavinogenic species in terms of their ability to overproduce and secrete riboflavin, whereas *E. cymbalariae* is not an overproducer (Sengupta and Chandra 2011).

Furthermore, the 8.7 Mb *A. gossypii* genome provided compelling evidence for whole-genome duplication in the *S. cerevisiae* lineage. This could be established based on the 2:1 mapping of *S. cerevisiae* loci to blocks of *A. gossypii* genes (Dietrich *et al.*, 2004). Similar 2:1 assignments have subsequently been reported for other unduplicated yeast genomes as well (Dujon *et al.* 2004; Kellis *et al.*, 2004). Using evolutionary genomics, we sought to close the gap between whole-genome duplication in one branch of the *Saccharomyces* lineage giving rise to a modern 12 Mb genome of *S. cerevisiae* and *A. gossypii* with one of the smallest genomes of a free living eukaryote in another. To this end, we determined the complete genome sequence of *E. cymbalariae* and annotated the predicted gene models. In a comparative approach, we elucidated the relationship of *E. cymbalariae* with the reconstructed yeast ancestor established by the Wolfe group and the *A. gossypii* genome (Dietrich *et al.*, 2004; Gordon *et al.*, 2009).

## Materials and Methods

### Strains and media

*Eremothecium cymbalariae* strain DBVPG #7215 was sequenced. *E. cymbalariae* is the type strain of the genus *Eremothecium* and was identified by Borzi in 1888 (Borzi 1888).

Cells were grown using complete media (1% yeast extract, 1% peptone, 2% dextrose).

### Sequencing strategy

*E. cymbalariae* genomic DNA was prepared according to Guthrie and Fink (1991). Instead of enzymatic digestion of the cell wall, grinding in liquid nitrogen was chosen. The DNA was prepared for next-generation sequencing at LGC Genomics (Berlin, Germany). Sequencing generated 1,000,000 reads on a 454 Roche GS FLX Titanium Sequencer. With an average of 400 bases per read, a total of ∼400 Mb of high-quality sequence data corresponding to a 40× coverage of the *E. cymbalariae* genome was produced. Primary assembly of the genome data resulted in 209 contigs, the largest of which was >500 kb. The average contig size was 45 kb.

Next, a fosmid library with a standard insert size of 30 kb was generated based on the pCC2FOS vector (Epicenter Biotechnologies, Madison, WI). From this library, paired-end reads of 750 fosmids were generated. Mapping these reads to the original contigs resulted in the generation of 35 supercontigs or scaffolds. Gaps within these scaffolds were closed by primer walking on the fosmid clones.

Remaining gaps between supercontigs were closed by generating and sequencing PCR products. Gap closure was completed in parallel with genome annotation to make use of syntenic relationships between the *E. cymbalariae* genome and the genome of *A. gossypii* or *S. cerevisiae*, or by inferring reciprocal translocations. This procedure yielded eight contigs corresponding to the eight chromosomes of *E. cymbalariae*.

### Annotation of the *E. cymbalariae* genome

The eight chromosomes of the *E. cymbalariae* genome were annotated and submitted to the EMBL/GenBank Data Libraries with accession numbers CP002497–CP002504. The mitochondrial genome has not been retrieved. Available fragments of the mitochondrial genome did not assemble the full mitochondrial genome at his stage. There is one region on CHR4 in the *E. cymbalariae* genome that carries the rDNA repeats (supporting information, Figure S3).

The *E. cymbalariae* genome was compared with the *A. gossypii* and *S. cerevisiae* genomes available from AGD (Ashbya Genome Database, http://agd.vital-it.ch/index.html) and SGD (Saccharomyces Genome Database, http://www.yeastgenome.org) using local BLAST tools (available at http://blast.ncbi.nlm.nih.gov). Using top-ranked hits, this produced a draft annotation of the *E. cymbalariae* genome. Fine annotation of the *E. cymbalariae* genome used syntenic relationships to *A. gossypii* and *S. cerevisiae*, as well as conservation of gene order with the reconstructed ancestral yeast genome. *E. cymbalariae* ORFs that did not occur in either of the genomes used for comparison were searched against the nonredundant dataset of NCBI. There are, however, 44 hypothetical ORFs for which no homolog could be identified. The identification of introns was mainly based on their conservation within homologous genes.

The current annotation was reinvestigated intensively to provide an as correct as possible initial characterization of the *E. cymbalariae* genome. Identification of *SPO16* as Ecym_2083a was based on synteny. However, this gene contains an intron, and the weak similarity of EcymSpo16 with its *A. gossypii* homolog made us miss this gene during the extensive annotation period; it was found only by specifically searching for this ZMM-family protein in *E. cymbalariae*. This clarifies how inevitable changes and the annotation of newly identified genes within the current nomenclature will be dealt with. EcymSPO16/Ecym_2083a can be found on CHR2 between the genes Ecym_2083 and Ecym_2084.

## Results/Discussion

### Sequencing of *E. cymbalariae*

**Figure 1:**
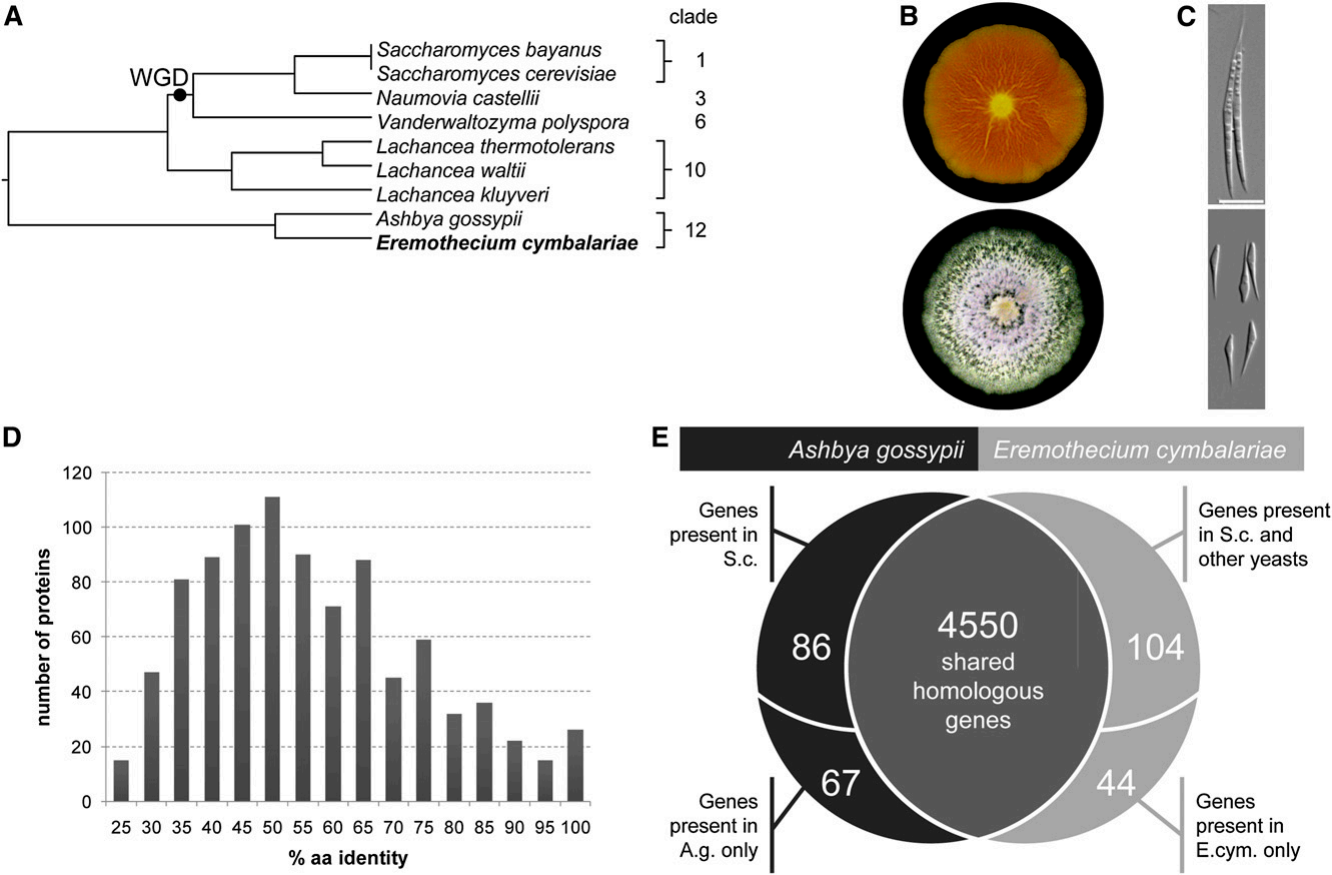
Comparison of *Eremothecium cymbalariae* with *Ashbya gossypii*. (A) Phylogenetic tree of selected species from the *Saccharomyces* complex based on 18S rDNA. The dot indicates the whole-genome duplication (WGD). Clade assignments are according to Kurtzman and Robnett (2003). (B) Fluffy aerial mycelial growth of *E. cymbalariae* compared with compact mycelial growth of the riboflavin overproducer *A. gossypii* (wrinkles indicate areas of strong hyphal adhesion). (C) Morphology of spores of *A. gossypii* (top) and *E. cymbalariae* (bottom). (D) Distribution of amino acid identities of all *E. cymbalariae* protein coding genes compared with the *A. gossypii* protein set. (E) Venn diagram indicating the distribution of homologous *vs.* non-homologous genes in both genomes. Genes occurring either in *A. gossypii* or *E. cymbalariae* may share homologs in *S. cerevisiae* or other yeasts as indicated.

*E. cymbalariae* was isolated and first described in 1888 by Borzi as a novel and very interesting fungus because of its systematic position (Borzi 1888). The genus name refers to the characteristic sporangia formed scarcely and in a solitary fashion at the tip of aerial hyphae. *Eremothecium* species today are grouped into clade 12 of the *Saccharomyces* complex according to Kurtzman and Robnett (2003) (Figure 1A). Both *E. cymbalariae* and *A. gossypii* are filamentous fungi that branch dichotomously at hyphal tips and thus can easily be distinguished from the dimorphic representatives in this genus, *Holleya sinecauda* and *E. coryli (*Schade *et al.*, 2003; Gastmann *et al.*, 2007). However, *E. cymbalariae* generates a fluffy aerial mycelium unique in this genus and in contrast to synnemata formed by *A. gossypii*. Additionally, *E. cymbalariae* mycelia are white, indicating a lack of riboflavin overproduction obvious from the yellow-colored *A. gossypii* mycelium (Figure 1B). *E. cymbalariae* forms only scarce sporangia, whereas *A. gossypii* produces abundant sporangia and spores. *E. cymbalariae* and *A. gossypii* spores differ in size and shape (Figure 1C). Whereas *E. cymbalariae* spores are on average 13 µm long and can be readily separated from each other, *A. gossypii* spores are more than twice as long and clump together via terminal filaments (Figure 1C). This has hampered efforts to set up mutagenic screens with *A. gossypii* because of the inability to isolate single spores. Interestingly, spore morphology of *A. gossypii* resembles that of the dimorphic relative *E. coryli* (Gastmann *et al.*, 2007).

We sequenced the *E. cymbalariae* genome with up to 40× coverage, and on the basis of the 9.7 Mb complete genome sequence, we annotated 4712 genes, which is very similar to the 4718 genes of *A. gossypii* and the 4824 genes of *Schizosaccharomyces pombe* (Dietrich *et al.*, 2004, Wood *et al.*, 2002). Details of the sequencing of the *E. cymbalariae* genome (accession numbers CP002497–CP002504) and supporting information are available in *Materials and Methods* and Table S1. The conservation of protein sequences between syntenic homologs of *E. cymbalariae* and *A. gossypii* varies considerably, ranging from 20 to 100%. Highly conserved genes include, for example, ribosomal proteins (Figure 1D). On average, there is a 60% identity between *E. cymbalariae* and *A. gossypii* proteins. This was surprising, as it indicates quite some evolutionary distance. This divergence is in a range of the *S. cerevisiae* and *Kluyveromyces lactis* species pair. For comparison, in *Saccharomyces sensu stricto* species, the level of protein identity is >85% (Cliften *et al.*, 2001). Especially the set of *Eremothecium* proteins that share only a low level of sequence identity may be informative of rapidly evolving proteins or processes that may warrant further study. On the other hand, 97% of all protein-encoding genes of *E. cymbalariae* share a homolog in *A. gossypii*. This leaves about 150 genes in each of the genomes not shared between *E. cymbalariae* and *A. gossypii* (Figure 1E). In the initial annotation of the *Ashbya* genome, ∼250 genes were found that do not have a homolog in *S. cerevisiae*. Comparing this set with the *E. cymbalariae* gene set indicates that most of these so-called NOHBYs (no homology in baker’s yeast) actually share a homolog in both *Eremothecium* species.

### Mapping centromeric loci to *E. cymbalariae* chromosomes

**Figure 2:**
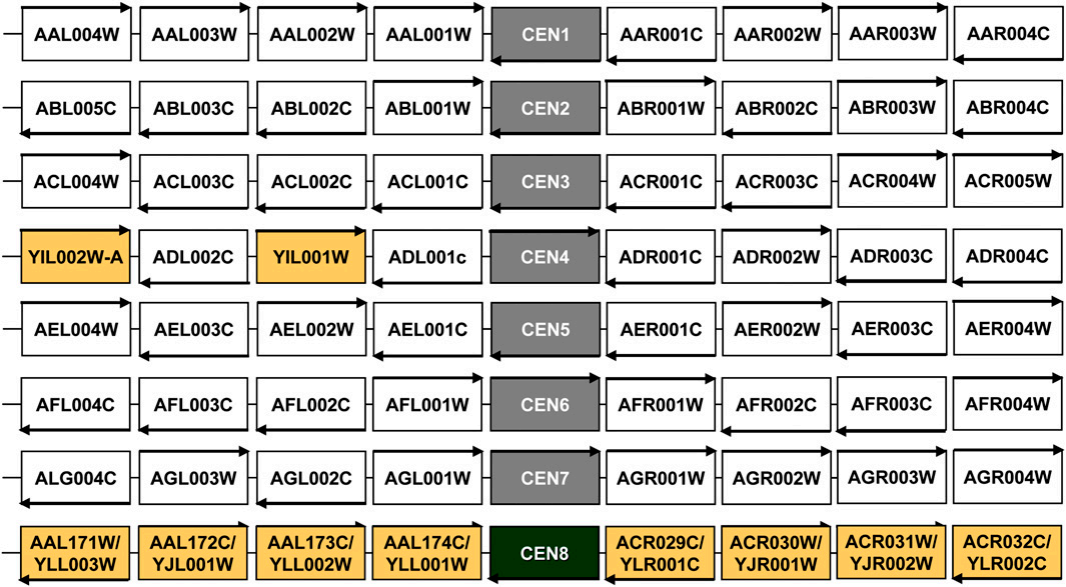
Chromosome assignment for all *E. cymbalariae* chromosomes based on synteny. Chromosomes 1 to 7 were assigned based on conserved centromere regions between *E. cymbalariae* and *A. gossypii. A. gossypii* systematic nomenclature is used. Arrows indicate transcriptional orientation of genes. Arrows for centromeres indicate orientation of Centromeric DNA Elements (CDEI-CDEII-CDEIII). *E. cymbalariae* centromeric region of CHR4 contains two genes that are homologous to *S. cerevisiae* genes but are absent in *A. gossypii*. *CEN8* was identified based on synteny to *S. cerevisiae* genes at centromere loci CHRX and CHRXII. *CEN8* has been eliminated in *A. gossypii*, and the chromosomal arms have been translocated to CHR1 and CHR3, respectively.

We mapped the completed genome of *E. cymbalariae* to eight contigs corresponding to eight chromosomes, in contrast to only seven chromosomes in *A. gossypii*. Using syntenic gene arrangements between *E. cymbalariae* and *A. gossypii*, we were able to identify seven *E. cymbalariae* centromeres, which were designated *CEN1* to *CEN7*, and the corresponding chromosomes, CHR1 to CHR7, respectively. This differs from the annotation in *A. gossypii*, which numbered the chromosomes according to size, with CHR1 being the smallest. *E. cymbalariae CEN8* was identified based on synteny to *S. cerevisiae* centromere loci on CHRX and CHRXII (Figure 2). The arrangement of the *E. cymbalariae CEN8* locus indicates that in *A. gossypii* both chromosomal arms of the ancestral CHR8 were translocated to two positions on other *A. gossypii* chromsomes, *i.e.* the telomeres of *A. gossypii* chromosomes I and III, with concomitant loss of *CEN8* but without any loss of genes. This mechanism of reduction in chromosome numbers is contrasted by all other cases in the *Saccharomyces* complex, in which telomere-to-telomere fusion of two chromosomes with subsequent loss of one of the centromeres was suggested to be predominant (Gordon *et al.*, 2011). Deviations from the number of eight chromosomes of the yeast ancestor have been found in pre–whole-genome duplication yeasts, *e.g.* *Kluyveromyces lactis* (six chromosomes) and *Zygosaccharomyces rouxii* (seven chromosomes). In post-WGD species, there are also deviations from 16 chromosomes, such as in *Candida glabrata* and *Naumovia castellii* (Dujon *et al.*, 2004; Gordon *et al.*, 2009, 2011; Souciet *et al.* 2009). This indicates that chromosome reduction was not the speciation event that distinguished the *Eremothecium* lineage from the other pre-WGD clades of the *Saccharomyces* complex, as eight ancestral chromosomes can still be found in *E. cymbalariae*, although a reduction to seven chromosomes in the genome of the *A. gossypii* ancestor could have separated this lineage from *E. cymbalariae*.

Point-like centromeres appeared already in the common ancestor of pre- and post-WGD species. Thus, centromeric DNA elements, CDEI, CDEII, and CDEIII are found to be highly conserved in the *Saccharomyces* complex (Gordon *et al.*, 2011). There is strong conservation between *E. cymbalariae* and *A. gossypii* centromeres, as could be expected of two closely related species. In particular, the CDEII region is ∼160 bp and thus twice as large as in *S. cerevisiae* (Figure S1).

### Comparison of telomere repeats in *Eremothecium*

**Figure 3:**
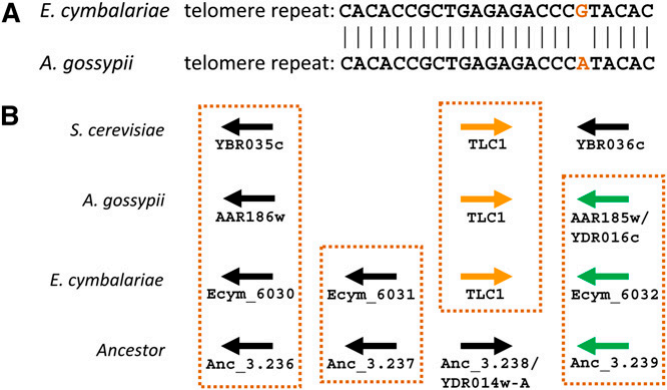
Identification of telomere repeats in *E. cymbalariae*. (A) Based on the identification of 9 out of 16 telomeres, the *E. cymbalariae* telomere repeat structure could be inferred. The 24 bp repeat of *E. cymbalariae* differs at a single position from the telomere repeat of *A. gossypii*. (B) In *S. cerevisiae*, the RNA component of telomerase is encoded by *TLC1*. This feature is located between YBR035C and YBR036C on CHRII. The *E. cymbalariae* and *A. gossypii TLC1* genes are found at syntenic positions. *E. cymbalariae TLC1* is located on CHR6 and additionally shares a gene with the deduced ancestor that is not found in *A. gossypii* or *S. cerevisiae*.

We identified 9 out of 16 telomeres of the *E. cymbalariae* genome. Thus, the *E. cymbalariae* genome currently lacks sequences at 7 chromosomal ends. With the available sequence information, we could determine the telomere repeat of *E. cymbalariae* as a 24 bp repeat sequence. It is characterized by tandem duplications of the sequence (CACACCGCTGAGAGACCCGTACAC)n. This repeat sequence differs from the telomere repeat of *A. gossypii* by a single base (Figure 3A). The only nontelomere copy of these repeats is in the putative *EcymTLC1* gene, which represents the RNA template component of telomerase. We identified this feature based on synteny to the ancestral *TLC1* locus and also via comparison with *S. cerevisiae* and *A. gossypii* (Figure 3B).

Even though our assembled sequence does not reach into telomere repeats, in seven cases a comparison with the reconstructed ancestor indicates that the terminal genes in this reconstructed ancestral genome were identified also in *E. cymbalariae*. Thus, even though the reconstruction of the ancestral genome is preliminary due to the more frequent recombination events at telomere loci, we believe that based on our high sequence coverage we may not have missed a large number of genes.

*E. cymbalariae*, like *A. gossypii*, does not harbor repetitive sequence elements such as *S. cerevisiae* Y′ elements at subtelomere regions. In other fungal model systems, such as *Magnaporthe oryzae* and *Ustilago maydis*, telomere-linked helicase gene families have been described, which are encoded by genes bearing similarity to the helicase genes of Y′ elements (Farman 2007).

In *S. cerevisiae*, these subtelomeric regions are hotspots of variation (Louis and Vershinin 2005). *S. cerevisiae* has accumulated several gene families at telomere regions. Particularly, genes of the *SUC* (invertase), *MAL* (maltose utilization), *MEL* (melibiose utilization), and *FLO* (flocculation) families, which are important for the fermentative life style of yeast, are clustered at telomeres (Teunissen and Steensma 1995).

Dispersal of these genes among different *S. cerevisiae* telomeres has been facilitated by rearrangements at chromosome ends. In the *A. gossypii* genome, several duplicated genes can be found at telomere loci [*e.g.* *JEN1* on the right arm of chromosome I (TEL1R) and the left arm of chromosome II (TEL2L), *OPT2* on TEL3L and TEL4L], and tandem duplications of telomere-associated genes can be found (*e.g.* a *OAF1* triplication near TEL4R or the distribution of mating-type loci at TEL4R and TEL5R) (Table S3B and see below). This provides evidence for sequence amplification by chromosomal rearrangements in *A. gossypii*, which we did not observe in *E. cymbalariae*.

### CHR1 harbors three mating-type cassettes in *E. cymbalariae*

We currently lack understanding of a sexual cycle in *A. gossypii. A. gossypii* produces spores that carry a single haploid nucleus. This feature is particularly useful in isolating homokaryotic mutants. Spores can germinate into a mycelium that can produce spores itself without requiring a mating partner. Thus *A. gossypii* may be homothallic (Wendland and Walther 2005). The *A. gossypii* strain that was sequenced was found to harbor three identical mating-type cassettes containing MAT**a** information.

**Figure 4:**
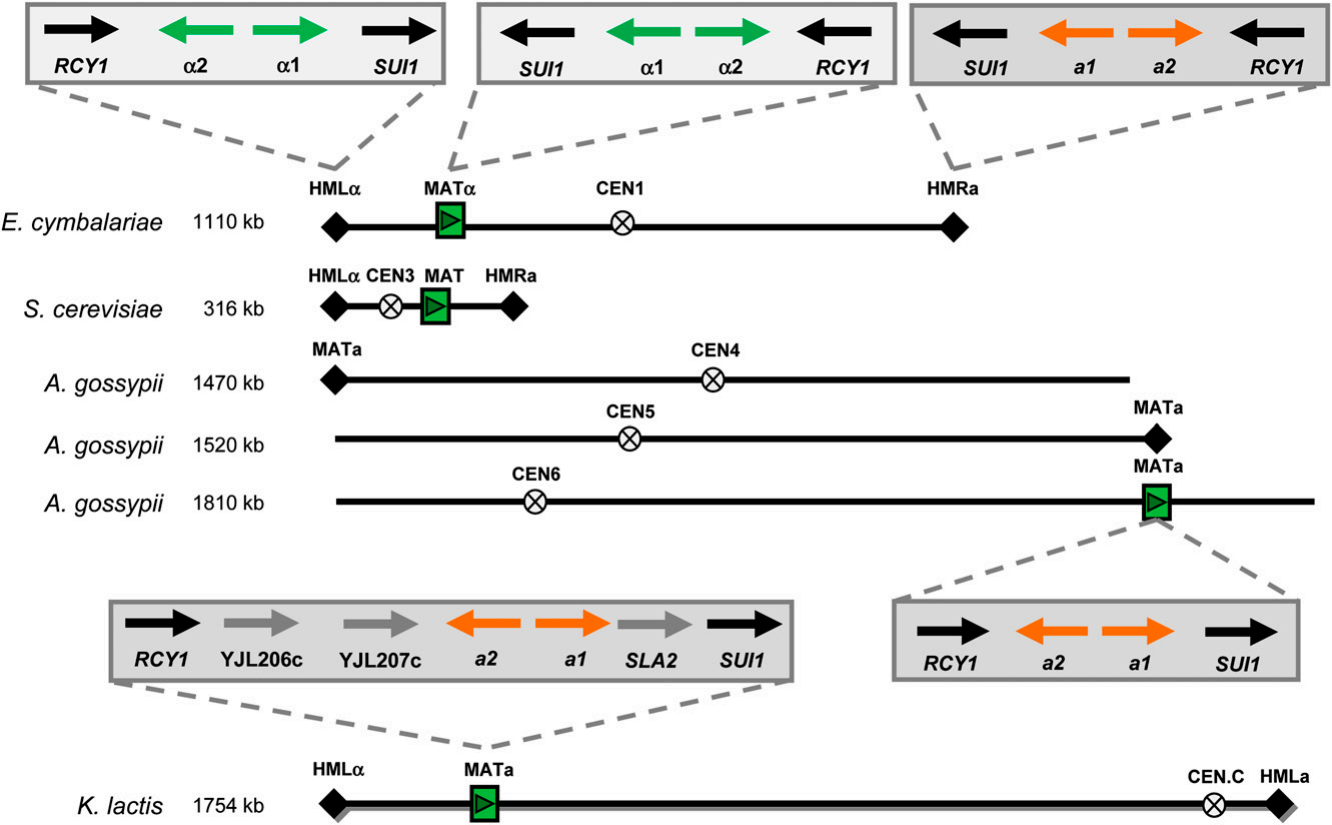
Comparative organization of mating-type loci and conserved flanking genes in *Saccharomycetes*. *MAT***a** loci are boxed in gray, *MATα* loci in yellow. *E. cymbalariae* shares the chromosomal arrangement of a *MAT* locus and two cassettes (*HMLα* and *HMR***a**) with *S. cerevisiae* and *K. lactis*. Genes flanking the mating-type loci are conserved between *E. cymbalariae* and *A. gossypii*. Both species have lost three genes (*S. cerevisiae* homologs of YJL206c, YJL207c, and *SLA2*) found at the *K. lactis MAT* locus. *MAT***a** on *A. gossypii* CHR6 is in a region of block synteny with the *E. cymbalariae MATα* on CHR1. Both telomeres of *A. gossypii* CHR6 are known and do not harbor additional mating-type cassettes.

In *S. cerevisiae*, mating type is determined by the expression of *MAT****a*** or *MATα* mating-type genes residing at the *MAT* locus (Figure 4). The active mating-type locus is on CHRIII of *S. cerevisiae*. At the telomeres of CHRIII, silent mating-type cassettes HMLα and HMR**a** are located. These loci can serve as templates for mating-type switching induced by *HO*-mediated DSB formation at the *MAT* locus (Haber 1998). In the *A. gossypii* and *E. cymbalariae* genome [as in other preduplication genomes, with the potential exception of *K. lactis* (Fabre *et al.*, 2005)], there is no homolog of the Ho-endonuclease. We found that *E. cymbalariae* harbors a conserved array of mating-type loci on CHR1 as found in *S. cerevisiae* CHRIII and in *K. lactis* (Figure 4). *E. cymbalariae MAT****a*** encodes two genes, *MAT***a***1* and *MAT****a****2*, whereas *MAT*α encodes *MAT*α*1* and *MAT*α*2*. The genes flanking the *MAT* loci are conserved between *E. cymbalariae* and *A. gossypii*. We recently analyzed several components of the mating pheromone signal transduction cascade in *A. gossypii* and showed that deletion of, for example, *AgSTE2* and *AgSTE3* does not inhibit sporulation. Deletion of the downstream transcription factor *AgSTE12*, on the other hand, resulted in increased sporulation. We also noted differences in genes encoding the *A. gossypii* α-factor pheromone. In *S. cerevisiae* and *E. cymbalariae*, there are two genes each encoding pre-proproteins containing several repeats of the mature respective α-factors. In *A. gossypii*, one of the homologous MFα genes does not contain a mature α-factor peptide, whereas the second gene harbors only a single peptide (Wendland *et al.*, 2011). Thus, there are striking differences between *E. cymbalariae* and *A. gossypii* concerning the organization of mating-type loci and in the genes involved in pheromone signaling (see also below). This actually makes *E. cymbalariae* an interesting model to investigate sexual reproduction in the filamentous *Eremothecium* species (*e.g.* concerning mating-type switching, pheromone signaling, and meiosis).

### Analysis of GC content in *Eremothecium* species

A unique feature of the *A. gossypii* genome within the *Saccharomyces* complex is its high GC content of 52% (Dietrich *et al.*, 2004). In contrast, we found that the GC content of *E. cymbalariae* with only 40% is similar to that of *S. cerevisiae* (38.3%) and other hemiascomycetous yeasts. Generally, the GC content around centromere regions (and at some telomeres) is lower than average in both *E. cymbalariae* and *A. gossypii* (Figure S2). A decrease in GC around centromere loci was noted also for other species. This may facilitate the identification of centromeres in species that do not have conserved point-like centromere structure (Gordon *et al.*, 2011). It was also noted that directional mutational pressure could result in evolutionary changes of GC content (Sueoka 1988). Recently it was suggested that gene conversion (*i.e.* the repair of double-strand breaks, DSB) is GC-biased in many eukaryotes including mammals (Duret and Galtier 2009; Galtier, *2003*; Marais 2003). Interestingly, in the yeast *Lachancea kluyveri*, the 1 Mb left arm of chromosome C shows a significantly higher GC content (52.9%) compared with the rest of the genome (40.4%), which may have been caused by an ancient hybridization event or by progressive GC content increase similar to a GC-biased repair. It was also noted that this arm is delayed in replication compared with the rest of the genome (Payen *et al.*, 2009)

We analyzed GC profile in *E. cymbalariae* and *A. gossypii* using a chromosome scale sliding window approach (GC profile available at http://tubic.tju.edu.cn/GC-Profile/; Gao and Zhang, 2006). For all chromosomes of both species, we could establish a GC-poor trough at centromere loci (one example for each species is shown in Figure S3). These regions of reduced GC content include at least 15 kb around the centromeres. Other areas with reduced GC content can be found at telomeres. Interestingly, in *A. gossypii* we found another GC coldspot on CHR4 between 827451 and 847198. This region contains 10 genes in *A. gossypii* that are in a syntenic block conserved between the reconstructed ancestor, *E. cymbalariae*, and *A. gossypii*. The functions of homologs of these genes have been analyzed in *S. cerevisiae*: six of these genes are essential; two are involved in cell cycle progression and sporulation; one in endocytosis; and for one, the function is unknown (Table S2). The homologous *E. cymbalariae* region also has a decreased GC content of 38.3% compared with 40.4% for the whole CHR8. This may suggest that GC coldspots mark regions of chromosomes harboring important genetic elements that may be adversely affected by GC-biased recombination. Conversely, this suggests that GC hotspots may pinpoint regions of frequent recombination, which may have altered genes in those loci in a species-specific manner. More refined functional analyses of such regions, therefore, seem warranted.

### Comparison of *Eremothecium* genomes

The genome of *E. cymbalariae* is 9.7 Mb and thus ∼900 kb larger than that of *A. gossypii* (excluding rDNA repeats; see Figure S3 and Table 1). This size difference is not based on a difference in gene number, as both *Eremothecium* species have about 4720 genes each. Gene density in *E. cymbalariae* is similar to that in *S. cerevisiae* with 2 kb/gene. The *A. gossypii* genome is more compact and gene density is <1.9 kb/gene. The size reduction of about 10% in the *A. gossypii* genome is due largely (>80%) to the streamlining of inter-ORF regions and far less to the extent of shortening ORFs.

**Table 1:** Comparison of *E. cymbalariae* and *A. gossypii* genomes

	*E. cymbalariae*	*A. gossypii*
Chromosomes	8	7
Genome size without rDNA	9671 kb	8765 kb
Genes	4711	4726
Gene density	2.05 kb/gene	1.86 kb/gene
Gene coding DNA	7118 kb	6970 kb
Gene coding DNA	73.62%	79.52%
Introns	232	226
tRNAs	143	192
GC content	40,3%	51,8%
TY3 transposon	1	—

We found one copy of a TY3-Gypsy transposon in *E. cymbalariae*, which does not occur in *A. gossypii*. There are remnants of a potential TY3 transposon on *Ashbya* CHRI and CHRIII and AGL264w may resemble a bacterial transposase (Dietrich *et al.*, 2004). Sites of transposon excision (LTRs or sigma elements) are found throughout the *E. cymbalariae* genome, suggesting that this transposon has been active (Table S1). Almost all *E. cymbalariae* genes that share homologs in *A. gossypii* or the reconstructed yeast ancestor are arranged in syntenic blocks. Orphan genes in both species are often associated with tRNAs or may be remnants of recombination or transposition events.

We adopted a systematic nomenclature for the *E. cymbalariae* genome that assigns each gene the species identifier “Ecym,” which is separated by an underscore from a four-digit number indicating the chromosomal location and assigning an ORF number. ORFs are numbered consecutively starting from the left telomere. Thus, Ecym_1001 designates the first ORF on chromosome 1 next to the left telomere, and similarly, Ecym_1541 will indicate the 541^st^ ORF (last ORF) on CHR1 counted from the left telomere. In this way, we annotated the complete genome. We took into account neither the position of the centromere on a chromosome nor the transcriptional orientation of an ORF. The identification of introns in *E. cymbalariae* was mainly based on their conservation within homologous genes. The intron splice rules for *E. cymbalariae* (5′-GT…TACTAAC…AG-3′) are conserved with those of *A. gossypii* and *S. cerevisiae* (Dietrich *et al.*, 2004, Spingola *et al.*, 1999). This led to the identification and annotation of 232 usually small putative introns. The average intron size is 173 bp. Most of the introns are located near the 5′ end of ORFs, as was also found in *A. gossypii* and *S. cerevisiae*. This small number of introns is in line with those observed in other fungi of the *Saccharomyces* complex. There are only a small number of genes in which putative introns differ between *E. cymbalariae* and *A. gossypii*. These occasions, therefore, indicate intron loss in the species without introns. With more and more genomes and transcriptomes available, a comprehensive map of ancestral introns can be compiled in the future.

To identify tRNA genes, tRNAscan was used (available at http://lowelab.ucsc.edu/ tRNAscan-SE/; Schattner *et al.*, 2005). This resulted in the annotation of 143 tRNA genes. This number is comparable to the 275 tRNAs in the *S. cerevisiae* genome when taking the WGD into account. *A. gossypii*, on the other hand, has 192 annotated tRNAs. This suggests an increase in tRNA genes in *A. gossypii*. Only four sets of tandemly duplicated tRNAs are present in the *A. gossypii* genome. Comparisons of blocks of syntenic gene arrangement between *A. gossypii* and *S. cerevisiae* have indicated that endpoints of synteny clusters are often marked by tRNAs (Dietrich *et al.*, 2004). This suggests that recombination via tRNAs as the only major source of repetitive elements in the *A. gossypii* genome resulted in the amplification of tRNA genes in Ashbya. Similarly, genome evolution can be driven by transposon-mediated recombination (Garfinkel 2005; Mieczkowski *et al.*, 2006). In *E. cymbalariae*, the primary insertion site of its TY3 transposon is next to tRNA genes, as was described for *S. cerevisiae* (Chalker and Sandmeyer 1992). This makes it difficult to distinguish between TY-mediated and tRNA-mediated genome evolution in *E. cymbalariae*.

### Genome evolution in pre-WGD species

In *A. gossypii*, 21 loci contain tandem duplications of two to four genes (Dietrich *et al.*, 2004). This limited amount of duplication does not allow for large-scale subfunctionalization. Yet, for the tandem duplication of the *RHO1* gene, it could be shown that changing a single position in the switch I region allows for a different regulation of these Rho proteins (Köhli *et al.*, 2008). In comparison, we found only eight of these tandem duplications to be conserved in *E. cymbalariae* (Table S3A). Several tandem duplicated *A. gossypii* genes are located at telomeres and are either not present or not duplicated in *E. cymbalariae*. This suggests species-specific changes and variability at telomeric positions in *A. gossypii*, which we have not found in *E. cymbalariae*.

**Figure 5:**
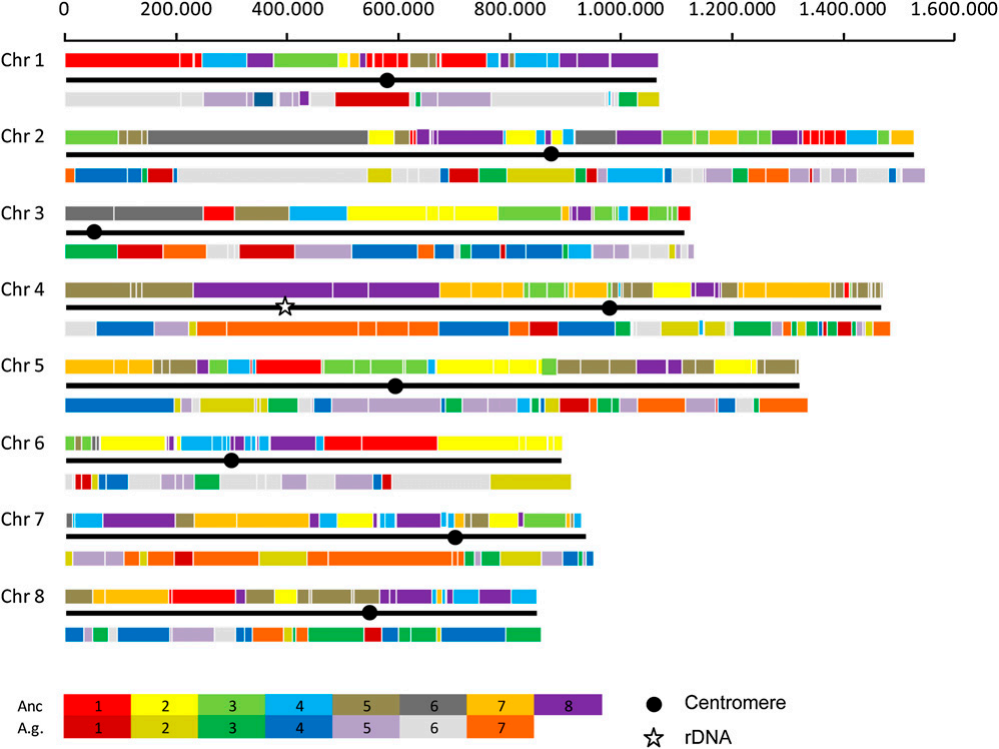
Genomic block synteny between the *E. cymbalariae* genome and *A. gossypii* and the reconstructed yeast ancestor. The colored blocks indicate regions of the reconstructed ancestral yeast genome (upper row) or the *A. gossypii* genome (bottom row). Colors correspond to the eight CHRs of the yeast ancestor and to the seven CHRs of *A. gossypii*, respectively. Scale indicates lengths of the *E. cymbalariae* chromosomes (bp); the dot corresponds to the centromere region of each chromosome; and the star indicates the rDNA locus. The rDNA locus in *A. gossypii* is ∼400 kb (Wendland *et al*., 1999). The additional DNA at the rDNA locus will make CHR4 the largest chromosome in *E. cymbalariae*.

We investigated the synteny relationships between the *E. cymbalariae* genome to both the yeast ancestor and *A. gossypii* (Figure 5). The *E. cymbalariae* genome can be mapped to the ancestor with 252 blocks of synteny and to *A. gossypii* with 229 blocks, with an average block length of 36.8 kb and 40.8 kb, respectively (Table 2). In contrast, the *A. gossypii* genome was previously mapped to 271 blocks with the yeast ancestor (Gordon *et al.*, 2009). Half of the *E. cymbalariae* genome size is represented in 40 blocks in the yeast ancestor and in only 38 blocks in *A. gossypii*. The largest blocks of synteny map to *E. cymbalariae* CHR2: 397 kb shared with the yeast ancestor and 340 kb with *A. gossypii*. There is also a large block of synteny flanking the conserved position of the rDNA repeats on *E. cymbalariae* CHR4, encompassing 251 kb in the yeast ancestor and 236 kb in *A. gossypii* (in addition to the rDNA repeats). Conservation of blocks is greater between *Eremothecium* species at centromeric loci. Yet, the large number of synteny blocks indicates an unexpectedly divergent genome evolution in both *Eremothecium* species. Once the genomes of other *Eremothecium* species become available, especially from the branch containing the dimorphic representatives, it may be possible to reconstruct an ancestral genome for the *Eremothecium* clade. For example, CHR6 of the reconstructed yeast ancestor is distributed to only seven blocks in the *E. cymbalariae* genome, two each on CHR2, CHR3, and CHR6, and one telomeric locus on CHR7. This allows the recapitulation of the evolution of this ancestral chromosome in *E. cymbalariae* by inferring only four inversions and translocations (three reciprocal and one telomeric) each.

**Table 2:** Number of syntenic blocks of the *E. cymbalariae* genome shared with the reconstructed ancestor and *A. gossypii*

*E. cymbalariae* Chromosome	Number of Blocks in the Ancestral Genome	Number of Blocks in the *A. gossypii* Genome
CHR1	**26**	23
CHR2	**38**	38
CHR3	**24**	28
CHR4	**42**	38
CHR5	**38**	35
CHR6	**32**	22
CHR7	**29**	22
CHR8	**23**	23
**Total**	**252**	**229**
**Average block length**	**36.8 kb**	**40.8 kb**

### Comparative genomics reveals species-specific evolutionary changes

Comparison of the *E. cymbalariae* and *A. gossypii* gene sets revealed that each species harbors around 150 genes that are absent in the other. Further analysis of these subsets of genes indicated that 86 *A. gossypii* genes absent in *E. cymbalariae* have homologs in *S. cerevisiae* and that 104 *E. cymbalariae* genes absent in *A. gossypii* share homologs in either *S. cerevisiae* or other related yeasts (Figure 1D, Table S4, and Table S5). The remaining genes are species-specific genes that were annotated based on their ORF lengths above cutoff. In *E. cymbalariae*, these genes contain ORFs from 300 to 450 bp in length. Lack of certain genes in an *Eremothecium* genome thus can be used to deduce species-specific evolutionary steps. These findings may then spur further functional analyses in either *A. gossypii* or *E. cymbalariae* to identify corresponding phenotypes in mutants in which such a gene has been deleted.

Three major differences involving the lack of pathways of genes between *E. cymbalariae* and *A. gossypii* were found. First, *E. cymbalariae* harbors a set of eight genes, termed ZMM proteins (*ZIP1–4*, *MER3*, *MSH4*, *MSH5*, and *SPO16*), which in *S. cerevisiae* were shown to facilitate meiotic crossovers (Lynn *et al.*, 2007). *A. gossypii* has lost most of these genes and has retained only a *ZIP1* homolog. This loss of ZMM family members in *A. gossypii* is puzzling. Combined with our recent analysis on the pheromone signaling cascade, this observation lets us question the existence of a sexual cycle (particularly one requiring mating and meiosis) in *A. gossypii* (Wendland *et al.*, 2011). On the other hand, with *E. cymbalariae* we now have an *Eremothecium* model in which we can analyze processes of mating-type switching, the role of MAT**a**/α for cell-type–specific gene expression, the role of pheromone signaling, and meiosis and sporulation.

Second, the *E. cymbalariae* genome does not encode a member of the *MNN1* gene family of α-1,3-mannosyltransferases. This family of five genes is present in both *S. cerevisiae* and *A. gossypii* and is required for adding the fourth and fifth mannose residue to a linear chain of *O*-glycosylated proteins (Romero *et al.*, 1999; Lussier *et al.*, 1999). Cell-cell adhesion via flocculins in *S. cerevisiae* requires binding of mannose residues (Goossens and Willaert, *2010*; Bauer *et al.*, 2010; Verstrepen and Klis 2006). Remarkably, the mycelium of *E. cymbalariae* and particularly the aerial hyphae show a penetrant lack of cell-cell adhesion, in contrast to the strong adhesion of *A. gossypii* hyphae. Therefore, *E. cymbalariae* may be a promising fungal model system to study adhesion, particularly with respect to the role of individual flocculation genes in protein-protein–mediated cell adhesion.

Third, in the *E. cymbalariae* genome we found a lack of genes belonging to the Ehrlich pathway of fusel acids/fusel alcohol production from amino acids. Key genes of this pathway include the transcriptional activator Aro80 and two downstream target genes *ARO9* and *ARO10* driving the first two steps of transamination and decarboxylation of amino acids to produce fusel aldehydes. Conversion of these aldehydes into either fusel acids or fusel alcohols is mediated by *ALD* and *ADH* gene families encoding aldehyde dehydrogenases or alcohol dehydrogenases, respectively (Hazelwood *et al.*, 2008). The *ARO9*, *ARO10*, and *ARO80* genes are not encoded in the *E. cymbalariae* genome. In addition, the *PDC5* gene, which encodes an alternative decarboxylase, the aldo-keto reductase *YPR1*, and *PDR12*, which encodes an ABC transporter involved in the export of fusel acids, are absent in *E. cymbalariae*. *A. gossypii* encodes all of these genes, with the exception of *ARO9*.

Fusel alcohol production has an impact on morphogenesis in *S. cerevisiae* as, for example, isoamyl alcohol, phenylethanol, and tryptophol can induce pseudohyphal growth and stimulate flocculation (Kern *et al.*, 2004; Chen and Fink, 2006,). Deletion of *ARO8*, *ARO9*, or *ARO80* renders *S. cerevisiae* cells defective in haploid invasive growth and diploid pseudohyphal formation. These compounds, therefore, can act as quorum-sensing molecules. Similar systems are utilized in other yeast species. In *Candida albicans*, for example, farnesol leads to the inhibition of filamentation at high cell densities (Hornby *et al.*, 2001).

Analysis of the flavor profiles of *A. gossypii* and *E. cymbalariae* are currently underway. *A. gossypii* produces far more aromatic compounds than *E. cymbalariae*, evident from a very fruity flavor. *A. gossypii* may produce these compounds to attract insects as part of its dispersal strategy. *E. cymbalariae* is rather unimpressive in terms of flavor production. Interestingly, lack of Ehrlich pathway genes can also be observed in *Kluyveromyces waltii*, which lacks, for example, the *ARO80* transcription factor according to the Yeast Gene Order Browser information (Byrne and Wolfe 2005).

### Perspectives

Our study demonstrates the power of comparative genomics to elucidate the evolutionary history of closely related species. The genome of *E. cymbalariae* harbors several features in common with the reconstructed yeast ancestor that have been lost in *A. gossypii*. In particular, we found the presence of eight chromosomes, similar gene densities, and an overall low GC content. Furthermore, in *E. cymbalariae* as in other yeasts, a mating-type locus and a cassette organization including silent cassettes at both telomeres can be found on one dedicated chromosome. In addition, we found a TY3 transposon and ZMM family genes required for meiotic crossovers in *E. cymbalariae*, which are absent from *A. gossypii*. With these characteristics, *E. cymbalariae* marks a missing link in the evolution from a yeast ancestor to *A. gossypii*.

Several *Eremothecium*-specific traits were discovered in addition to the filamentous growth mode, including the telomeric repeats and conserved flanks at mating-type loci. We speculate that the *E. cymbalariae*–specific loss of α-1,3-mannosyltransferases and of key Ehrlich pathway genes may contribute to the lack of cell-cell adhesion observed in this fungal species. On the other hand, specific evolutionary steps in *A. gossypii*, which have yet to be uncovered, have turned this species into an oversporulator in comparison with *E. cymbalariae* and into an overproducer of riboflavin. Both features are linked, as riboflavin production is increased at the end of the growth phase concomitantly with sporulation (Stahmann *et al.*, 2001).

Comparative genomics is a very powerful tool to reveal specific features of closely related species not only in, for example, the *Saccharomyces* complex but also in other systems as demonstrated for the *Ustilago maydis/Sporisorium reilianum* species pair (Schirawski *et al.*, 2010).

## Supplementary Material

Supporting Information
