# Supplementary material for: Genome Evolution in the Eremothecium Clade of the Saccharomyces Complex Revealed by Comparative Genomics
Source: G3 (Bethesda). 2011 Dec 1;1(7):539–48. doi: 10.1534/g3.111.001032 (PMC3276169; doi:10.1534/g3.111.001032)
Supplement: Supporting Information [file supp_1_7_539__index.html]

Supporting Information 

# Genome Evolution in the *Eremothecium* Clade of the *Saccharomyces* Complex Revealed by Comparative Genomics

## Supporting Information for Wendland and Walther, 2011

**Files in this Data Supplement:**

- Supporting Information - Figures S1-S3 and Tables S1-S5 (PDF, 780 KB)
- Figure S1 - Centromere DNA Element (CDE) conservation in *Eremothecium* (PDF, 232 KB)
- Figure S2 - Analysis of GC-content on a chromosomal scale (PDF, 52 KB)
- Figure S3 - Comparison of the rDNA repeat units of *A. gossypii* and *S. cerevisiae* (Wendland *et al.*, 1999) with the rDNA-repeat of *E. cymbalariae* (PDF, 60 KB)
- Table S1 - *E. cymbalariae* genome summary (PDF, 52 KB)
- Table S2 - One *A. gossypii* GC-cold spot harbors many essential genes (PDF, 48 KB)
- Table S3 - A) Comparison of tandem duplicated genes of *A. gossypii* with *E. cymbalariae* homologs B) Duplicated genes at telomeric loci in Ashbya gossypii (PDF, 132 KB)
- Table S4 - Genes found in *E. cymbalariae* but not in *A. gossypii* (PDF, 152 KB)
- Table S5 - Genes found in *A. gossypii* but not in *E. cymbalariae* (PDF, 136 KB)
