# Supplementary material for: Genome Evolution in the Eremothecium Clade of the Saccharomyces Complex Revealed by Comparative Genomics
Source: G3 (Bethesda). 2011 Dec 1;1(7):539–48. doi: 10.1534/g3.111.001032 (PMC3276169; doi:10.1534/g3.111.001032)
Supplement: Supporting Information [file supp_1.7.539_001032SI.pdf]

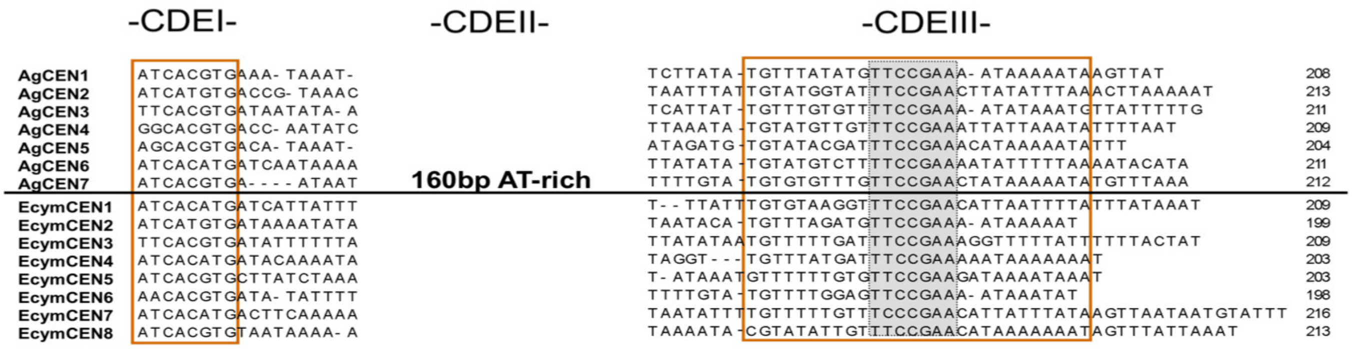

Figure S1 Centromere DNA Element (CDE) conservation in *Eremothecium*. CDEI is characterised by the palindrome CACGTG. CDEII is a 160bp AT-rich region, whereas CDEIII has a central conserved motif of TTCCGAA in a 25bp core sequence. These point-like centromeres differ from S. cerevisiae in the length of the CDEII element.

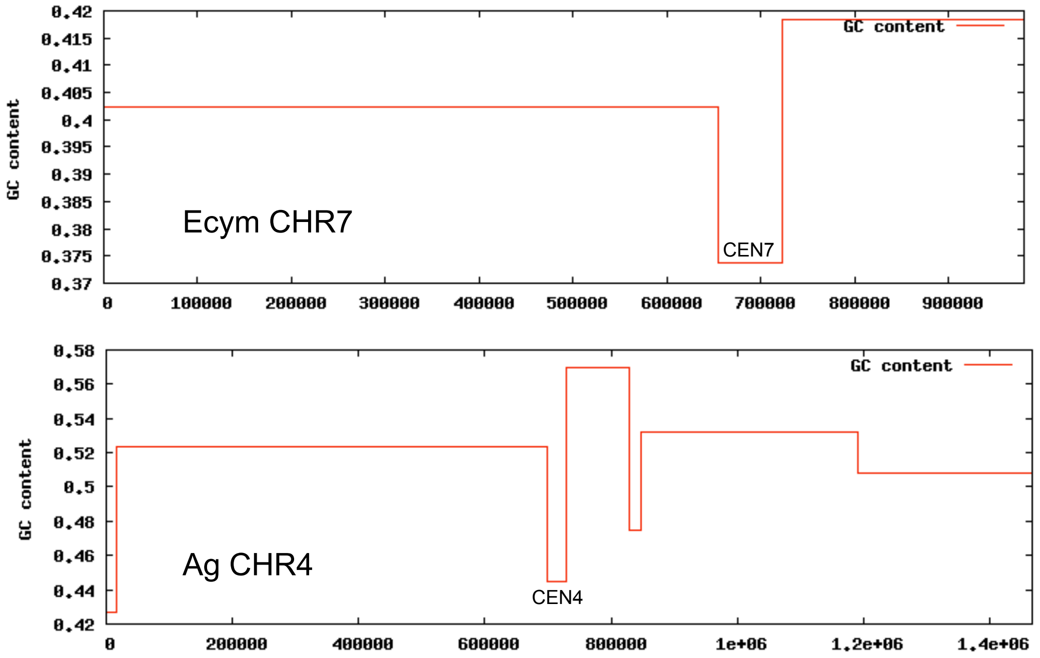

Figure S2 Analysis of GC-content on a chromosomal scale. GC-profiles were generated using the online tool at http://tubic.tju.edu.cn/GC-Profile/. (Gao and Zhang, 2006). Upper graph shows *E. cymbalariae* CHR 7 with its lower GC content around CEN7 (located at 695316-695514). Lower graph shows *A. gossypii* CHR4. CEN4 is located at 708288-708477. Two more regions with reduced GC content are found on this chromosome. One at the left telomere. The other between 827451-847198. For details on the genes in that region confer to Table S2.

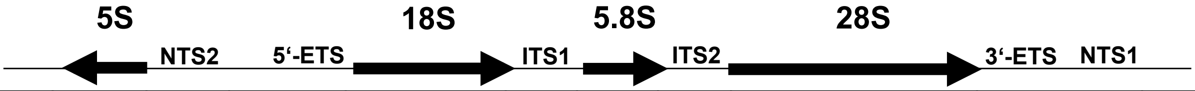

**Ag**	121	734	618	1795	207	158	210	3390	964	**8197bp**
	**100%**			**99%**		**97%**		**97%**		
**Ecym**	121	696	625	1796	206	158	210	3391	858	**8061bp**
	**100%**			**97%**		**92%**		**94%**		
**Sc**	121	1243	701	1800	361	158	232	3396	1125	**9137bp**

Figure S3 Comparison of the rDNA repeat units of *A. gossypii* and *S. cerevisiae* (Wendland *et al.*, 1999) with the rDNA-repeat of *E. cymbalariae*. A single repeat unit (not to scale) is shown starting with the 5S rRNA gene that is divergently transcribed from the precursor 35S pre-RNA (running from 5'-ETS to 3'-ETS). This precursor contains the 18S, 5.8S, and 25S rRNA genes separated by internal transcribed spacers (ITS1, ITS2) and flanked by external transcribed spacers (5'ETS, 3'ETS). The 5S rRNA gene and the 35S pre-rRNA are separated by non-transcribed spacers (NTS1, NTS2). Length of the individual mature rRNAs and spacer regions is indicated. Using pairwise comparisons % identity of rRNAs between *E. cymbalariae* and *A. gossypii* and between *E. cymbalariae* and *S. cerevisiae* is indicated.

Table S1 ***E. cymbalariae*** **genome summary**

CHR	lenths (bp)	coding[1] (bp)	% encoding	Features	gene density/kb	ORFs	tRNAs	LTRs	#genes with introns[2]	#of introns	total length of introns (bp)	telomeric repeats	Accession number
1	1.110.245	807.030	72.10	541	2.05	530	11	7	33	36	6525	0	**CP002497**
2	1.601.921	1.172.903	72.82	823	1.95	799	24	13	31	33	6329	1	**CP002498**
3	1.193.613	876.657	73.16	622	1.99	598	24	16	27	28	3364	1	**CP002499**
4	1.541.034 +rDNA repeat	1.152.108	74.34	792	1.95	767	25	7	38	39	6554	2	**CP002500**
5	1.385.851 +TY3	1.040.071	74.75	679	2.04	662	17	5	20	20	4127	2	**CP002501**
6	959.278	687.803	71.31	491	1.95	475	16	7	20	20	3713	1	**CP002502**
7	980.088	717.760	72.62	480	2.04	466	14	4	31	31	5988	0	**CP002503**
8	897.456	663.863	73.58	427	2.10	415	12	7	24	25	3471	2	**CP002504**
total	**9.669.486**	**7.118.195**	**73.62**	**4855**	**2.05**	**4712**	**143**	**66**	**224**	**232**	**40.071**	**9/16**	

1) The values in coding (bp) and % encoding refer to protein encoding genes, while gene density calculations also include tRNA genes.
2) The number of genes with introns refers to protein coding genes. There are also 44 tRNAs with introns.

Table S2 One *A. gossypii* GC-cold spot harbors many essential genes[1]

Ancestor	*E. cymbalariae*	*A. gossypii*	*S. cerevisiae*	Function in *S. cerevisiae*
Anc_7.332	Ecym_8.082	ADR068W	YLR210W (CLB4)	B-type cyclin
Anc_7.331		absent	YDL154W (MSH5)	meiotic recombination
	Ecym_8.081			
Anc_7.330	Ecym_8.080	ADR069C	YDL153C (SAS10)	essential
Anc_7.329	Ecym_8.079	ADR070W	YDL150W (RPC53)	essential
Anc_7.328	Ecym_8.078	ADR071W	YDL149W (APG9)	Sporulation absent; viability decreased
Anc_7.327	Ecym_8.077	ADR072C	YDL148C (NOP14)	essential
Anc_7.326	Ecym_8.076	ADR073W	YDL147W (RPN5)	essential
Anc_7.325	Ecym_8.075	ADR074C	YLR211C	
Anc_7.324	Ecym_8.074	ADR075W	YDL146W	actin cytoskeleton defects; endocytosis decreased
Anc_7.323	Ecym_8.073	ADR076C	YLR212C (TUB4)	essential
Anc_7.322	Ecym_8.072	ADR077C	YDL145C (COP1)	essential

1. This is one example of a GC coldspot. A more detailed anaylsis of GC hotspots and GC coldspots will be presented elsewhere.

Table S3 Comparison of tandem duplicated genes of *A. gossypii* with *E. cymbalariae* homologs

Ag[1,2,3]	Ag	Ag	Ag	Sc	Ecym	Ecym	Ecym
AAL179W[3]	AAL178W			YJL079C (PRY1)<br>YKR013W (PRY2)<br>YJL078C (PRY3)	Ecym_6325	Ecym_6326	
ABL189W	ABL188W			YDL237W	Ecym_6472	Ecym_6471	
**ABR025C**	**ABR026C**	**ABR027C**	**ABR028C**	**YKL096W (CWP1)**	**Ecym_2423**		
ABR182W	ABR183W			YPR165W (RHO1)	Ecym_7211	Ecym_7212	
**ABR246W**[3]	**ABR247W**	**ABR248W**	**ABR249W**	**YIR035C/YIR036C**	**none**		
**ACL202W**[3]	**ACL201W**	**ACL200W**		**YMR238W (DFG5)**	**none**		
ACR098C	ACR099C			YPL129W (ANC1)<br>YOR213C (SAS5)	Ecym_8318	Ecym_8319	
**ACR143W**	**ACR144W**			**YPL154C (PEP4)**	**Ecym_2396**		
**ACR272C**[3]	**ACR273W**			**YKL096W (CWP1)**	**none**		
**ADL156C**	**ADL155C**			**YOL119C (MCH4)**	**Ecym_2054**		
**ADR081C**	**ADR082C**			**YLR215C (CDC123)**	**Ecym_8069**		
**ADR336C**	**ADR337C**			**YNR055C (HOL1)**	**Ecym_5082**		
ADR403C	ADR404C	ADR405C		YAL051W (OAF1)	Ecym_5017	Ecym_5016	Ecym_5015

			YOR363C (PIP2)		
**AER452C**[3]	**AER453C**	**AER454C**	**YJR107W**	**none**	
AFL095W	AFL092C		YHR211W (FLO5)	Ecym_2200	Ecym_2201
**AFR262C**	**AFR263C**		**YGL246C (RAI1)**	**Ecym_2600**	
AGL352W[3]	AGL351W		YMR307W (GAS1)	Ecym_5666	Ecym_5665
**AGL326W**	**AGL325W**		**YJL172W (CPS1)**	**Ecym_7172**	
AGR038C	AGR039C		YDR046C (BAP3)<br>YBR068C (BAP2)	Ecym_2662	Ecym_2663
AGR188W	AGR189W		YDR227W (SIR4)	Ecym_4126	Ecym_4125
**AGR405C**[3]	**AGR406C**		**YCL057W (PRD1)**	**Ecym_1011**	

1) Ag = *Ashbya gossypii*; Sc = *Saccharomyces cerevisiae*; Ecym = *Eremothecium cymbalariae*

2) Genes printed in bold are absent or not duplicated in *E. cymbalariae* – no duplications in *E. cymbalariae* were detected that do not occur in *A. gossypii*.

3) Positioned at telomeric loci in *A. gossypii*

Table S3b   Duplicated genes at telomeric loci in Ashbya gossypii

Ashbya gossypii gene	*Saccharomyces cerevisiae* gene	Location in *A. gossypii*
ACR082c	YAL018c	TEL3R
ACR083c		TEL3R
ADL027w		Internal
ADR122c		Internal
ADR403c	YAL051w	TEL4R
ADR404c	(OAF1)	TEL4R
ADR405c		TEL4R
AGR405c	YCL057w	TEL7R
AGR406c	(PRD1)	TEL7R
ABR228c	YFL041w	TEL2R
ADR239w	(FET5)	internal
AAR183c	YIL014w	TEL1R
ABR245c	(MNT3)	TEL2R
ADR020c		Internal
AEL345w		TEL5L
AFL235w		TEL6L
AFR301c	YIL159w	Internal
AGL364c	(BNR1)	TEL7L
ACL203c	YIL166c	TEL3L
AER444w		TEL5R
AFR229c		Internal
AGR235w		Internal
ABR246w	YIR035c	TEL2R
ABR247w		TEL2R
ABR248w		TEL2R
ABR249w		TEL2R
ACR171c		internal
AER445c	YJR076c	TEL5R
AFR436c	(CDC11)	Internal
ABR159c	YJR107w	Internal
ACL114w		Internal
AER452c		TEL5R
AER453c		TEL5R
AER454c		TEL5R

ABR025c	YKL096w	Internal
ABR026c	(CWP1)	Internal
ABR027c		Internal
ABR028c		Internal
ACR272c		TEL3R
ACR273w		TEL3R
ADL398c		TEL4L
AAR192c	YKL217w	TEL1R
ABL210c	(JEN1)	TEL2L
AFR333w		Internal
ACL200w	YMR238w	TEL3L
ACL201w	(DFG5)	TEL3L
ACL202w		TEL3L
AFR530w		Internal
AGL351w	YMR307w	TEL7L
AGL352w	(GAS1)	TEL7L
ABL123c	YPL154c	Internal
ACR143w	(PEP4)	Internal
ACR144w		Internal
AGR407c		TEL7R
ACL205c	YPR194c	TEL3L
ADL399c	(OPT2)	TEL4L
AGL027w		internal

Mating-type loci are excluded

Table S4 Genes found in *E. cymbalariae* but not in *A. gossypii*.

E. cymbalariae genes with homologs in *S. cerevisiae* that are absent from *A. gossypii*		
1	Ecym_1002	**YCL067C HMLALPHA2**
2	Ecym_1003	**YCL066W HMLALPHA1**
3	Ecym_1114	**YCL067C MATALPHA1**
4	Ecym_1115	**YCL066W MATALPHA2**
5	Ecym_1027	**YIR030C DCG1**
6	Ecym_1028	**YDR524C-B**
7	Ecym_1059	**YDR506C**
8	Ecym_1093	**YMR318C ADH6**
9	Ecym_1223	**YBR067C TIP1**
10	Ecym_1351	**YCR019W MAK32**
11	Ecym_1394	**YDL209C CWC2**
12	Ecym_1496	**YCR021C HSP30**
13	Ecym_2013	**YNL327W EGT2**
14	Ecym_2169	**YPL223C GRE1**
15	Ecym_2268	**YGL108C**
16	Ecym_2317	**YDR387C**
17	Ecym_2360	**YOR264w DSE3**
18	Ecym_2537	**YPL039W**
19	Ecym_2567	**YBR161W CSH1**
20	Ecym_2668	**YBR071W**
21	Ecym_2706	**YML107C PML39**
22	Ecym_2731	**YGL159W**
23	Ecym_2772	**YLR376C PSY3**
24	Ecym_3107	**YLR286C CTS1**
25	Ecym_3263	**YLR394W CST9/ZIP3**
26	Ecym_3264	**YDL124W**

27	Ecym_3364	**YOL105C WSC3**
28	Ecym_3399	**YMR090W**
29	Ecym_3408	**YKL084W HOT13**
30	Ecym_3569	**YEL040W UTR2**
31	Ecym_4001	**YER185W PUG1**
32	Ecym_4027	**YOR084W LPX1**
33	Ecym_4036	**YKR087C OMA1**
34	Ecym_4205	**YDR171W HSP42**
35	Ecym_4288	**YLR097C HRT3**
36	Ecym_4297	**YDR119W-A**
37	Ecym_4437	**YPR134W**
38	Ecym_4485	**YIL001W**
39	Ecym_4487	**YIL002W-A**
40	Ecym_4607	**YML113W DAT1**
41	Ecym_5140	**YBR047W FMP23**
42	Ecym_5363	**YOR087W YVC1**
43	Ecym_5427	**YNL115C**
44	Ecym_5553	**YDR072C IPT1**
45	Ecym_5557	**YDR061W**
46	Ecym_5577	**YGR110W CLD1**
47	Ecym_6004	**YGL251C HFM1/MER3**
48	Ecym_6006	**YGL249W ZIP2**
49	Ecym_6031	**YOR390W**
50	Ecym_6064	**YLR040C**
51	Ecym_6268	**YLR363C NMD4**
52	Ecym_7011	**YJR152W DAL5**
53	Ecym_7049	**YBR188C NTC20**
54	Ecym_7097	**YHR149C SKG6**
55	Ecym_7235	**YBL005W PDR3**

56	Ecym_7251	**YMR073C IRC21**
57	Ecym_7252	**YMR072W ABF2**
58	Ecym_7256	**YMR069W NAT4**
59	Ecym_7277	**YPR010C-A**
60	Ecym_7308	**YFL003C MSH4**
61	Ecym_7342	**YGL041W-A**
62	Ecym_7419	**YOR320C GNT1**
63	Ecym_8023	**YIL073C SPO22/ZIP4**
64	Ecym_8081	**YDL154W MSH5**
65	Ecym_8390	**YDR032C PST2**
66	Ecym_8420	**YHL038C CBP2**

***E. cymbalariae* genes absent from *S. cerevisiae* and *A. gossypii* but share homologs in other yeasts[1]**

67	Ecym_1007	**KLLA0C00462g**
68	Ecym_1012	**KLLA0C00594g**
69	Ecym_1082	**KLLA0C02233g**
70	Ecym_1420	**KLTH0H09306g**
71	Ecym_2062	**KLLA0E15620g**
72	Ecym_2283	**KLLA0B00495g**
73	Ecym_3096	**KLLA0A04521g**
74	Ecym_3372	**KLLA0E14806g**
75	Ecym_4528	**KLLA0E03377g**
76	Ecym_5045	**KLLA0F23221g**
77	Ecym_5162	**KLLA0B04224g**
78	Ecym_5211	**KLLA0E15356g**
79	Ecym_5262	**KLLA0C04433g**
80	Ecym_5281	**KLTH0C08624g**
81	Ecym_5338	**KLLA0F21164g**

82	Ecym_5415	**KLLA0F23573g**
83	Ecym_6130	**KLLA0A02871g**
84	Ecym_6199	**KLLA0C11539g**
85	Ecym_6395	**KLLA0D11066p**
86	Ecym_6433	**KLLA0D08624p**
87	Ecym_6440	**KLLA0F20856g**
88	Ecym_7076	**KLLA0E24442g**
89	Ecym_7169	**KLLA0E05544g**
90	Ecym_8149	**KLLA0D10395g**
91	Ecym_8160	**KLLA0E17039g**
92	Ecym_8221	**KLLA0F22198g**
93	Ecym_8354	**KLLA0D14399g ScYAP5/YAP7**
94	Ecym_1178	**KLLA0E23079g**
95	Ecym_2506	**KLTH0A06908g**
96	Ecym_2679	**KLTH0A00440g**
97	Ecym_5041	**KLTH0D01408g**
98	Ecym_7253	**KLTH0D06886g**
99	Ecym_7302	**KLTH0A02728g**
100	Ecym_4373	**Kpol_1063p1**
101	Ecym_3623	**NFIA_005660**
102	Ecym_3158	**SCOCAAB17122**
103	Ecym_3156	**ZYRO0G01870g**
104	Ecym_5064	**ZYRO0D04224g**

**Hypothetical *E. cymbalariae* genes without homolog in the databases**	
105	Ecym_1213
106	Ecym_1460
107	Ecym_2057
108	Ecym_2064

109	Ecym_2234
110	Ecym_2655
111	Ecym_2740
112	Ecym_2756
113	Ecym_2780
114	Ecym_3228
115	Ecym_3234
116	Ecym_3288
117	Ecym_3377
118	Ecym_3379
119	Ecym_3380
120	Ecym_3401
121	Ecym_3441
122	Ecym_3536
123	Ecym_4267
124	Ecym_4268
125	Ecym_4495
126	Ecym_4582
127	Ecym_4583
128	Ecym_4774
129	Ecym_5223
130	Ecym_5230
131	Ecym_5238
132	Ecym_5449
133	Ecym_5602
134	Ecym_6046
135	Ecym_6063
136	Ecym_6119
137	Ecym_6120

138	Ecym_6121
139	Ecym_6172
140	Ecym_6237
141	Ecym_6297
142	Ecym_6371
143	Ecym_6450
144	Ecym_7298
145	Ecym_7394
146	Ecym_7433
147	Ecym_7444
148	Ecym_8269

1) KLLA: *Kluyveromyces lactis;* KLTH: *Lachancea thermotolerans;* Kpol: *Vanderwaltozyma polyspora;* NFIA: *Neosartorya fischeri;* SCOC: *Schwanniomyces occidentalis;* ZYR: *Zygosaccharomyces rouxii*

Table S5 Genes found in *A. gossypii* but not in *E. cymbalariae*.

A. gossypii genes with homologs in *S. cerevisiae* that are absent from *E. cymbalariae*		
1	AAL068C	**YHR035W**
2	AAL112C	**YOR258W (HNT3)**
3	AAL128C	**YBR072W (HSP26)**
4	AAR033W	**YPR127W**
5	AAR084W	**YMR303C (ADH2)**
6	AAR086W	**YGR097W (ASK10)**
7	AAR129C	**YDR415C**
8	AAR183C	**YIL014W (MNT3)**
9	ABL084C	**YJR142W**
10	ABL090W	**YBR046C (ZTA1)**
11	ABL210C	**YKL217W (JEN1)**
12	ABR050W	**YMR012W (CLU1)**
13	ABR053C	**YMR015C (ERG5)**
14	ABR113C	**YLL055W**
15	ABR185W	**YCL057W (PRD1)**
16	ABR229C	**YJR159W (SOR1)**
17	ABR245C	**YIL014W (MNT3)**
18	ACL085C	**YNL092W**
19	ACL203C	**YIL166C**
20	ACL205C	**YPR194C (OPT2)**
21	ACR211W	**YDR380W (ARO10)**
22	ADL015C	**YNL104C (LEU4)**
23	ADL194W	**YOL141W (PPM2)**
24	ADL199W	**YML005W**
25	ADL258W	**YNL065W (AQR1)**
26	ADL328C	**YPR062W (FCY1)**
27	ADL362C	**YGR161C**
28	ADL392W	**YHR202W**
29	ADL397C	**YBR072W (HSP26)**
30	ADR020C	**YIL014W (MNT3)**
31	ADR079C	**YDL144C**
32	ADR119W	**YBR284W**
33	ADR142W	**YMR064W (AEP1)**
34	ADR199C	**YDR421W (ARO80)**
35	ADR205C	**YOR062C**

36	ADR229C	**YOR079C (ATX2)**
37	ADR237C	**YHL039W**
38	ADR286C	**YML004C (GLO1)**
39	ADR365W	**YOR337W (TEA1)**
40	AEL057C	**YCR023C**
41	AEL099W	**YDR338C**
42	AEL114C	**YJL163C**
43	AEL143W	**YCR028C (FEN2)**
44	AEL151C	**YJL190C (RPS22A)**
45	AEL171C	**YJL059W (YHC3)**
46	AEL345W	**YIL014W (MNT3)**
47	AER313C	**YDR452W (PPN1)**
48	AER338C	**YMR209C**
49	AER401W	**YDR368W (YPR1)**
50	AER409C	**YDR022C (CIS1)**
51	AER426C	**YIL137C**
52	AER444W	**YIL166C**
53	AER445C	**YJR076C (CDC11)**
54	AER459w	**YBR072W (HSP26)**
55	AFL158C	**YAL067C (SEO1)**
56	AFL160C	**YPL248C (GAL4)**
57	AFL204C	**YHR096C (HXT5)**
58	AFL235W	**YIL014W (MNT3)**
59	AFR045W	**YHR132C (ECM14)**
60	AFR206C	**YKL140W (TGL1)**
61	AFR228W	**YKR093W (PTR2)**
62	AFR322C	**YGR138C (TPO2)**
63	AFR333W	**YKL217W (JEN1)**
64	AFR415C	**YJR062C (NTA1)**
65	AFR437W	**YBR072W (HSP26)**
66	AFR506C	**YFL014W (HSP12)**
67	AFR548C	**YGL202W (ARO8)**
68	AFR561W	**YMR226C**
69	AFR633W	**YBR072W (HSP26)**
70	AFR675W	**YNL274C**
71	AFR726W	**YDR525W-A (SNA2)**
72	AGL027W	**YPR194C (OPT2)**
73	AGL049C	**YGR017W**
74	AGL050C	**YGR019W (UGA1)**

75	AGL087C	**YGL012W (ERG4)**
76	AGL142C	**YPL058C (PDR12)**
77	AGL143C	**YLR130C (ZRT2)**
78	AGL322W	**YBL039W-B**
79	AGL359C	**YER187W**
80	AGR108C	**YER176W (ECM32)**
81	AGR110W	**YER175C (TMT1)**
82	AGR235W	**YIL166C**
83	AGR250C	**YGR111W**
84	AGR297C	**YKR070W**
85	AGR329C	**YHR179W (OYE2)**
86	AGR407C	**YPL154C**

A. gossypii* genes absent from *S. cerevisiae* and *E. cymbalariae		
87	ABL004W	
88	ABL060C	
89	ABL062C	
90	ABL067C	
91	ABL157W	
92	ABL167C	
93	ABR042W	
94	ACL007C	
95	ACL049W	
96	ACL115W	
97	ACL133W	
98	ACL156W	
99	ACL204W	
100	ACR002C	
101	ACR141W	
102	ACR169C	
103	ADL011W	
104	ADL070C	
105	ADL112W	
106	ADL148C	
107	ADL359C	
108	ADL393W	
109	ADL400W	
110	ADR057W	
111	ADR100C	

112	ADR245W
113	ADR246C
114	ADR347C
115	AER099C
116	AER101W
117	AER160C
118	AER382W
119	AER455C
120	AFL013C
121	AFL028W
122	AFL126W
123	AFL137W
124	AFL164C
125	AFL233C
126	AFL234C
127	AFR048W
128	AFR070W
129	AFR079C
130	AFR164W
131	AFR170C
132	AFR288C
133	AFR311C
134	AFR476C
135	AFR622W
136	AFR740W
137	AFR742W
138	AFR746C
139	AGL037W
140	AGL048C
141	AGL084C
142	AGL146W
143	AGL277W
144	AGL309W
145	AGL366C
146	AGL367C
147	AGL368W
148	AGL369W
149	AGR013C
150	AGR055C

151	AGR090C
152	AGR124C
153	AGR404W
