# Supplementary material for: Genome Evolution in the Eremothecium Clade of the Saccharomyces Complex Revealed by Comparative Genomics
Source: G3 (Bethesda). 2011 Dec 1;1(7):539–48. doi: 10.1534/g3.111.001032 (PMC3276169; doi:10.1534/g3.111.001032)
Supplement: Supporting Information [file supp_1.7.539_FigureS1.pdf]

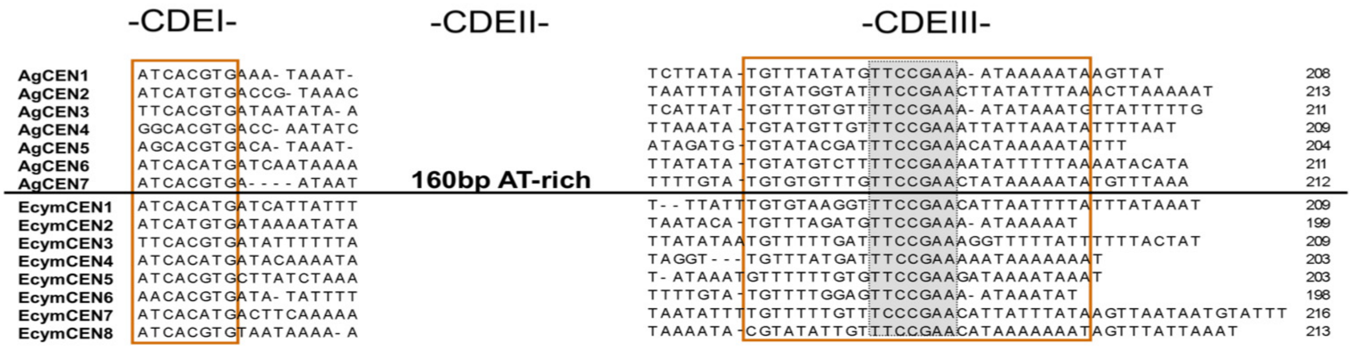

Figure S1 Centromere DNA Element (CDE) conservation in *Eremothecium*. CDEI is characterised by the palindrome CACGTG. CDEII is a 160bp AT-rich region, whereas CDEIII has a central conserved motif of TTCCGAA in a 25bp core sequence. These point-like centromeres differ from S. cerevisiae in the length of the CDEII element.
