# Supplementary material for: Genome Evolution in the Eremothecium Clade of the Saccharomyces Complex Revealed by Comparative Genomics
Source: G3 (Bethesda). 2011 Dec 1;1(7):539–48. doi: 10.1534/g3.111.001032 (PMC3276169; doi:10.1534/g3.111.001032)
Supplement: Supporting Information [file supp_1.7.539_FigureS2.pdf]

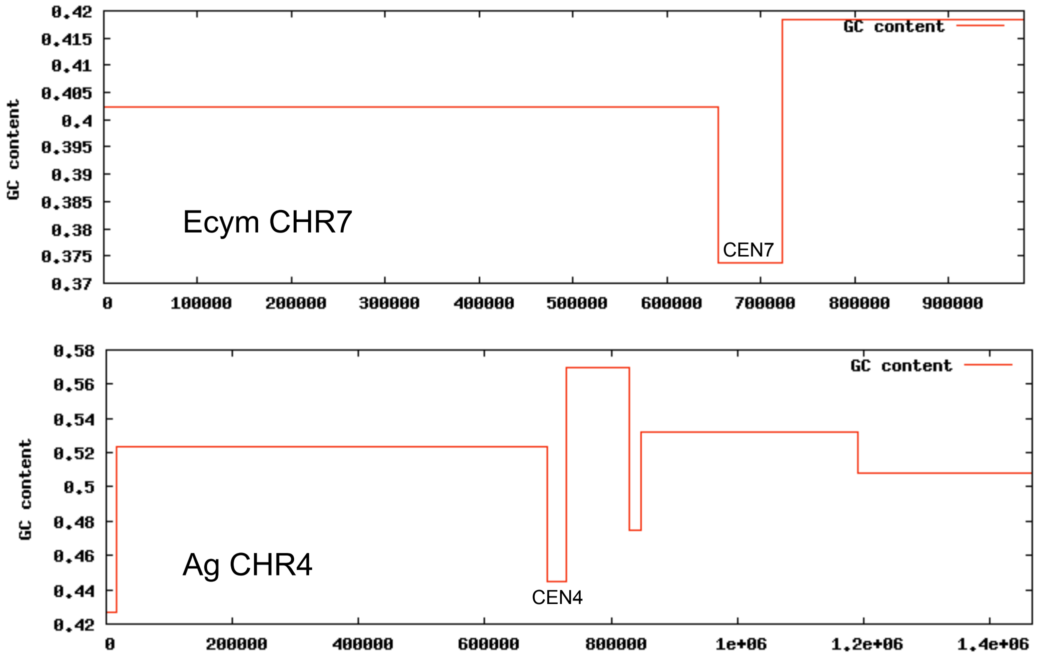

Figure S2 Analysis of GC-content on a chromosomal scale. GC-profiles were generated using the online tool at http://tubic.tju.edu.cn/GC-Profile/. (Gao and Zhang, 2006). Upper graph shows *E. cymbalariae* CHR 7 with its lower GC content around CEN7 (located at 695316-695514). Lower graph shows *A. gossypii* CHR4. CEN4 is located at 708288-708477. Two more regions with reduced GC content are found on this chromosome. One at the left telomere. The other between 827451-847198. For details on the genes in that region confer to Table S2.
