# Supplementary material for: Genome Evolution in the Eremothecium Clade of the Saccharomyces Complex Revealed by Comparative Genomics
Source: G3 (Bethesda). 2011 Dec 1;1(7):539–48. doi: 10.1534/g3.111.001032 (PMC3276169; doi:10.1534/g3.111.001032)
Supplement: Supporting Information [file supp_1.7.539_FigureS3.pdf]

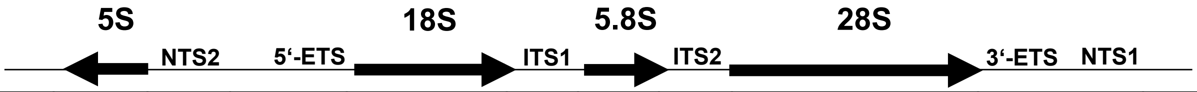

**Ag**	121	734	618	1795	207	158	210	3390	964	**8197bp**
	**100%**			**99%**		**97%**		**97%**		
**Ecym**	121	696	625	1796	206	158	210	3391	858	**8061bp**
	**100%**			**97%**		**92%**		**94%**		
**Sc**	121	1243	701	1800	361	158	232	3396	1125	**9137bp**

Figure S3 Comparison of the rDNA repeat units of *A. gossypii* and *S. cerevisiae* (Wendland *et al.*, 1999) with the rDNA-repeat of *E. cymbalariae*. A single repeat unit (not to scale) is shown starting with the 5S rRNA gene that is divergently transcribed from the precursor 35S pre-RNA (running from 5’-ETS to 3’-ETS). This precursor contains the 18S, 5.8S, and 25S rRNA genes separated by internal transcribed spacers (ITS1, ITS2) and flanked by external transcribed spacers (5’ETS, 3’ETS). The 5S rRNA gene and the 35S pre-rRNA are separated by non-transcribed spacers (NTS1, NTS2). Length of the individual mature rRNAs and spacer regions is indicated. Using pairwise comparisons % identity of rRNAs between *E. cymbalariae* and *A. gossypii* and between *E. cymbalariae* and *S. cerevisiae* is indicated.
