# Supplementary material for: Genome Evolution in the Eremothecium Clade of the Saccharomyces Complex Revealed by Comparative Genomics
Source: G3 (Bethesda). 2011 Dec 1;1(7):539–48. doi: 10.1534/g3.111.001032 (PMC3276169; doi:10.1534/g3.111.001032)
Supplement: Supporting Information [file supp_1.7.539_TableS1.pdf]

Table S1 ***E. cymbalariae*** **genome summary**

CHR	lenths (bp)	coding[1] (bp)	% encoding	Features	gene density/kb	ORFs	tRNAs	LTRs	#genes with introns[2]	#of introns	total length of introns (bp)	telomeric repeats	Accession number
1	1.110.245	807.030	72.10	541	2.05	530	11	7	33	36	6525	0	**CP002497**
2	1.601.921	1.172.903	72.82	823	1.95	799	24	13	31	33	6329	1	**CP002498**
3	1.193.613	876.657	73.16	622	1.99	598	24	16	27	28	3364	1	**CP002499**
4	1.541.034 +rDNA repeat	1.152.108	74.34	792	1.95	767	25	7	38	39	6554	2	**CP002500**
5	1.385.851 +TY3	1.040.071	74.75	679	2.04	662	17	5	20	20	4127	2	**CP002501**
6	959.278	687.803	71.31	491	1.95	475	16	7	20	20	3713	1	**CP002502**
7	980.088	717.760	72.62	480	2.04	466	14	4	31	31	5988	0	**CP002503**
8	897.456	663.863	73.58	427	2.10	415	12	7	24	25	3471	2	**CP002504**
total	**9.669.486**	**7.118.195**	**73.62**	**4855**	**2.05**	**4712**	**143**	**66**	**224**	**232**	**40.071**	**9/16**	

1) The values in coding (bp) and % encoding refer to protein encoding genes, while gene density calculations also include tRNA genes.
2) The number of genes with introns refers to protein coding genes. There are also 44 tRNAs with introns.
