# Supplementary material for: Genome Evolution in the Eremothecium Clade of the Saccharomyces Complex Revealed by Comparative Genomics
Source: G3 (Bethesda). 2011 Dec 1;1(7):539–48. doi: 10.1534/g3.111.001032 (PMC3276169; doi:10.1534/g3.111.001032)
Supplement: Supporting Information [file supp_1.7.539_TableS2.pdf]

Table S2 One *A. gossypii* GC-cold spot harbors many essential genes[1]

Ancestor	***E. cymbalariae***	***A. gossypii***	***S. cerevisiae***	**Function in *S. cerevisiae***
Anc_7.332	Ecym_8.082	ADR068W	YLR210W (CLB4)	B-type cyclin
Anc_7.331		absent	YDL154W (MSH5)	meiotic recombination
	Ecym_8.081			
Anc_7.330	Ecym_8.080	ADR069C	YDL153C (SAS10)	essential
Anc_7.329	Ecym_8.079	ADR070W	YDL150W (RPC53)	essential
Anc_7.328	Ecym_8.078	ADR071W	YDL149W (APG9)	Sporulation absent; viability decreased
Anc_7.327	Ecym_8.077	ADR072C	YDL148C (NOP14)	essential
Anc_7.326	Ecym_8.076	ADR073W	YDL147W (RPN5)	essential
Anc_7.325	Ecym_8.075	ADR074C	YLR211C	
Anc_7.324	Ecym_8.074	ADR075W	YDL146W	actin cytoskeleton defects; endocytosis decreased
Anc_7.323	Ecym_8.073	ADR076C	YLR212C (TUB4)	essential
Anc_7.322	Ecym_8.072	ADR077C	YDL145C (COP1)	essential

1. This is one example of a GC coldspot. A more detailed anaylsis of GC hotspots and GC coldspots will be presented elsewhere.
