# Supplementary material for: Genome Evolution in the Eremothecium Clade of the Saccharomyces Complex Revealed by Comparative Genomics
Source: G3 (Bethesda). 2011 Dec 1;1(7):539–48. doi: 10.1534/g3.111.001032 (PMC3276169; doi:10.1534/g3.111.001032)
Supplement: Supporting Information [file supp_1.7.539_TableS3.pdf]

Table S3 Comparison of tandem duplicated genes of *A. gossypii* with *E. cymbalariae* homologs

Ag[1,2,3]	Ag	Ag	Ag	Sc	Ecym	Ecym	Ecym
AAL179W[3]	AAL178W			YJL079C (PRY1)<br>YKR013W (PRY2)<br>YJL078C (PRY3)	Ecym_6325	Ecym_6326	
ABL189W	ABL188W			YDL237W	Ecym_6472	Ecym_6471	
**ABR025C**	**ABR026C**	**ABR027C**	**ABR028C**	**YKL096W (CWP1)**	**Ecym_2423**		
ABR182W	ABR183W			YPR165W (RHO1)	Ecym_7211	Ecym_7212	
**ABR246W**[3]	**ABR247W**	**ABR248W**	**ABR249W**	**YIR035C/YIR036C**	**none**		
**ACL202W**[3]	**ACL201W**	**ACL200W**		**YMR238W (DFG5)**	**none**		
ACR098C	ACR099C			YPL129W (ANC1)<br>YOR213C (SAS5)	Ecym_8318	Ecym_8319	
**ACR143W**	**ACR144W**			**YPL154C (PEP4)**	**Ecym_2396**		
**ACR272C**[3]	**ACR273W**			**YKL096W (CWP1)**	**none**		
**ADL156C**	**ADL155C**			**YOL119C (MCH4)**	**Ecym_2054**		
**ADR081C**	**ADR082C**			**YLR215C (CDC123)**	**Ecym_8069**		
**ADR336C**	**ADR337C**			**YNR055C (HOL1)**	**Ecym_5082**		
ADR403C	ADR404C	ADR405C		YAL051W (OAF1)	Ecym_5017	Ecym_5016	Ecym_5015

			YOR363C (PIP2)		
**AER452C**[3]	**AER453C**	**AER454C**	**YJR107W**	**none**	
AFL095W	AFL092C		YHR211W (FLO5)	Ecym_2200	Ecym_2201
**AFR262C**	**AFR263C**		**YGL246C (RAI1)**	**Ecym_2600**	
AGL352W[3]	AGL351W		YMR307W (GAS1)	Ecym_5666	Ecym_5665
**AGL326W**	**AGL325W**		**YJL172W (CPS1)**	**Ecym_7172**	
AGR038C	AGR039C		YDR046C (BAP3)<br>YBR068C (BAP2)	Ecym_2662	Ecym_2663
AGR188W	AGR189W		YDR227W (SIR4)	Ecym_4126	Ecym_4125
**AGR405C**[3]	**AGR406C**		**YCL057W (PRD1)**	**Ecym_1011**	

1) Ag = *Ashbya gossypii*; Sc = *Saccharomyces cerevisiae*; Ecym = *Eremothecium cymbalariae*

2) Genes printed in bold are absent or not duplicated in *E. cymbalariae* – no duplications in *E. cymbalariae* were detected that do not occur in *A. gossypii*.

3) Positioned at telomeric loci in *A. gossypii*

Table S3b   Duplicated genes at telomeric loci in Ashbya gossypii

Ashbya gossypii gene	*Saccharomyces cerevisiae* gene	Location in *A. gossypii*
ACR082c	YAL018c	TEL3R
ACR083c		TEL3R
ADL027w		Internal
ADR122c		Internal
ADR403c	YAL051w	TEL4R
ADR404c	(OAF1)	TEL4R
ADR405c		TEL4R
AGR405c	YCL057w	TEL7R
AGR406c	(PRD1)	TEL7R
ABR228c	YFL041w	TEL2R
ADR239w	(FET5)	internal
AAR183c	YIL014w	TEL1R
ABR245c	(MNT3)	TEL2R
ADR020c		Internal
AEL345w		TEL5L
AFL235w		TEL6L
AFR301c	YIL159w	Internal
AGL364c	(BNR1)	TEL7L
ACL203c	YIL166c	TEL3L
AER444w		TEL5R
AFR229c		Internal
AGR235w		Internal
ABR246w	YIR035c	TEL2R
ABR247w		TEL2R
ABR248w		TEL2R
ABR249w		TEL2R
ACR171c		internal
AER445c	YJR076c	TEL5R
AFR436c	(CDC11)	Internal
ABR159c	YJR107w	Internal
ACL114w		Internal
AER452c		TEL5R
AER453c		TEL5R
AER454c		TEL5R

ABR025c	YKL096w	Internal
ABR026c	(CWP1)	Internal
ABR027c		Internal
ABR028c		Internal
ACR272c		TEL3R
ACR273w		TEL3R
ADL398c		TEL4L
AAR192c	YKL217w	TEL1R
ABL210c	(JEN1)	TEL2L
AFR333w		Internal
ACL200w	YMR238w	TEL3L
ACL201w	(DFG5)	TEL3L
ACL202w		TEL3L
AFR530w		Internal
AGL351w	YMR307w	TEL7L
AGL352w	(GAS1)	TEL7L
ABL123c	YPL154c	Internal
ACR143w	(PEP4)	Internal
ACR144w		Internal
AGR407c		TEL7R
ACL205c	YPR194c	TEL3L
ADL399c	(OPT2)	TEL4L
AGL027w		internal

Mating-type loci are excluded
