# Supplementary material for: Genome Evolution in the Eremothecium Clade of the Saccharomyces Complex Revealed by Comparative Genomics
Source: G3 (Bethesda). 2011 Dec 1;1(7):539–48. doi: 10.1534/g3.111.001032 (PMC3276169; doi:10.1534/g3.111.001032)
Supplement: Supporting Information [file supp_1.7.539_TableS4.pdf]

Table S4 Genes found in *E. cymbalariae* but not in *A. gossypii*.

E. cymbalariae genes with homologs in *S. cerevisiae* that are absent from *A. gossypii*		
1	Ecym_1002	**YCL067C HMLALPHA2**
2	Ecym_1003	**YCL066W HMLALPHA1**
3	Ecym_1114	**YCL067C MATALPHA1**
4	Ecym_1115	**YCL066W MATALPHA2**
5	Ecym_1027	**YIR030C DCG1**
6	Ecym_1028	**YDR524C-B**
7	Ecym_1059	**YDR506C**
8	Ecym_1093	**YMR318C ADH6**
9	Ecym_1223	**YBR067C TIP1**
10	Ecym_1351	**YCR019W MAK32**
11	Ecym_1394	**YDL209C CWC2**
12	Ecym_1496	**YCR021C HSP30**
13	Ecym_2013	**YNL327W EGT2**
14	Ecym_2169	**YPL223C GRE1**
15	Ecym_2268	**YGL108C**
16	Ecym_2317	**YDR387C**
17	Ecym_2360	**YOR264w DSE3**
18	Ecym_2537	**YPL039W**
19	Ecym_2567	**YBR161W CSH1**
20	Ecym_2668	**YBR071W**
21	Ecym_2706	**YML107C PML39**
22	Ecym_2731	**YGL159W**
23	Ecym_2772	**YLR376C PSY3**
24	Ecym_3107	**YLR286C CTS1**
25	Ecym_3263	**YLR394W CST9/ZIP3**
26	Ecym_3264	**YDL124W**

27	Ecym_3364	**YOL105C WSC3**
28	Ecym_3399	**YMR090W**
29	Ecym_3408	**YKL084W HOT13**
30	Ecym_3569	**YEL040W UTR2**
31	Ecym_4001	**YER185W PUG1**
32	Ecym_4027	**YOR084W LPX1**
33	Ecym_4036	**YKR087C OMA1**
34	Ecym_4205	**YDR171W HSP42**
35	Ecym_4288	**YLR097C HRT3**
36	Ecym_4297	**YDR119W-A**
37	Ecym_4437	**YPR134W**
38	Ecym_4485	**YIL001W**
39	Ecym_4487	**YIL002W-A**
40	Ecym_4607	**YML113W DAT1**
41	Ecym_5140	**YBR047W FMP23**
42	Ecym_5363	**YOR087W YVC1**
43	Ecym_5427	**YNL115C**
44	Ecym_5553	**YDR072C IPT1**
45	Ecym_5557	**YDR061W**
46	Ecym_5577	**YGR110W CLD1**
47	Ecym_6004	**YGL251C HFM1/MER3**
48	Ecym_6006	**YGL249W ZIP2**
49	Ecym_6031	**YOR390W**
50	Ecym_6064	**YLR040C**
51	Ecym_6268	**YLR363C NMD4**
52	Ecym_7011	**YJR152W DAL5**
53	Ecym_7049	**YBR188C NTC20**
54	Ecym_7097	**YHR149C SKG6**
55	Ecym_7235	**YBL005W PDR3**

56	Ecym_7251	**YMR073C IRC21**
57	Ecym_7252	**YMR072W ABF2**
58	Ecym_7256	**YMR069W NAT4**
59	Ecym_7277	**YPR010C-A**
60	Ecym_7308	**YFL003C MSH4**
61	Ecym_7342	**YGL041W-A**
62	Ecym_7419	**YOR320C GNT1**
63	Ecym_8023	**YIL073C SPO22/ZIP4**
64	Ecym_8081	**YDL154W MSH5**
65	Ecym_8390	**YDR032C PST2**
66	Ecym_8420	**YHL038C CBP2**

E. cymbalariae **genes absent from** ***S. cerevisiae*** **and** ***A. gossypii*** **but share homologs in other yeasts**[1]

67	Ecym_1007	**KLLA0C00462g**
68	Ecym_1012	**KLLA0C00594g**
69	Ecym_1082	**KLLA0C02233g**
70	Ecym_1420	**KLTH0H09306g**
71	Ecym_2062	**KLLA0E15620g**
72	Ecym_2283	**KLLA0B00495g**
73	Ecym_3096	**KLLA0A04521g**
74	Ecym_3372	**KLLA0E14806g**
75	Ecym_4528	**KLLA0E03377g**
76	Ecym_5045	**KLLA0F23221g**
77	Ecym_5162	**KLLA0B04224g**
78	Ecym_5211	**KLLA0E15356g**
79	Ecym_5262	**KLLA0C04433g**
80	Ecym_5281	**KLTH0C08624g**
81	Ecym_5338	**KLLA0F21164g**

82	Ecym_5415	**KLLA0F23573g**
83	Ecym_6130	**KLLA0A02871g**
84	Ecym_6199	**KLLA0C11539g**
85	Ecym_6395	**KLLA0D11066p**
86	Ecym_6433	**KLLA0D08624p**
87	Ecym_6440	**KLLA0F20856g**
88	Ecym_7076	**KLLA0E24442g**
89	Ecym_7169	**KLLA0E05544g**
90	Ecym_8149	**KLLA0D10395g**
91	Ecym_8160	**KLLA0E17039g**
92	Ecym_8221	**KLLA0F22198g**
93	Ecym_8354	**KLLA0D14399g ScYAP5/YAP7**
94	Ecym_1178	**KLLA0E23079g**
95	Ecym_2506	**KLTH0A06908g**
96	Ecym_2679	**KLTH0A00440g**
97	Ecym_5041	**KLTH0D01408g**
98	Ecym_7253	**KLTH0D06886g**
99	Ecym_7302	**KLTH0A02728g**
100	Ecym_4373	**Kpol_1063p1**
101	Ecym_3623	**NFIA_005660**
102	Ecym_3158	**SCOCAAB17122**
103	Ecym_3156	**ZYRO0G01870g**
104	Ecym_5064	**ZYRO0D04224g**

**Hypothetical *E. cymbalariae* genes without homolog in the databases**	
105	Ecym_1213
106	Ecym_1460
107	Ecym_2057
108	Ecym_2064

109	Ecym_2234
110	Ecym_2655
111	Ecym_2740
112	Ecym_2756
113	Ecym_2780
114	Ecym_3228
115	Ecym_3234
116	Ecym_3288
117	Ecym_3377
118	Ecym_3379
119	Ecym_3380
120	Ecym_3401
121	Ecym_3441
122	Ecym_3536
123	Ecym_4267
124	Ecym_4268
125	Ecym_4495
126	Ecym_4582
127	Ecym_4583
128	Ecym_4774
129	Ecym_5223
130	Ecym_5230
131	Ecym_5238
132	Ecym_5449
133	Ecym_5602
134	Ecym_6046
135	Ecym_6063
136	Ecym_6119
137	Ecym_6120

138	Ecym_6121
139	Ecym_6172
140	Ecym_6237
141	Ecym_6297
142	Ecym_6371
143	Ecym_6450
144	Ecym_7298
145	Ecym_7394
146	Ecym_7433
147	Ecym_7444
148	Ecym_8269

1) KLLA: *Kluyveromyces lactis;* KLTH: *Lachancea thermotolerans;* Kpol: *Vanderwaltozyma polyspora;* NFIA: *Neosartorya fischeri;* SCOC: *Schwanniomyces occidentalis;* ZYR: *Zygosaccharomyces rouxii*
