# Supplementary material for: Genome Evolution in the Eremothecium Clade of the Saccharomyces Complex Revealed by Comparative Genomics
Source: G3 (Bethesda). 2011 Dec 1;1(7):539–48. doi: 10.1534/g3.111.001032 (PMC3276169; doi:10.1534/g3.111.001032)
Supplement: Supporting Information [file supp_1.7.539_TableS5.pdf]

Table S5 Genes found in *A. gossypii* but not in *E. cymbalariae*.

A. gossypii genes with homologs in *S. cerevisiae* that are absent from *E. cymbalariae*		
1	AAL068C	**YHR035W**
2	AAL112C	**YOR258W (HNT3)**
3	AAL128C	**YBR072W (HSP26)**
4	AAR033W	**YPR127W**
5	AAR084W	**YMR303C (ADH2)**
6	AAR086W	**YGR097W (ASK10)**
7	AAR129C	**YDR415C**
8	AAR183C	**YIL014W (MNT3)**
9	ABL084C	**YJR142W**
10	ABL090W	**YBR046C (ZTA1)**
11	ABL210C	**YKL217W (JEN1)**
12	ABR050W	**YMR012W (CLU1)**
13	ABR053C	**YMR015C (ERG5)**
14	ABR113C	**YLL055W**
15	ABR185W	**YCL057W (PRD1)**
16	ABR229C	**YJR159W (SOR1)**
17	ABR245C	**YIL014W (MNT3)**
18	ACL085C	**YNL092W**
19	ACL203C	**YIL166C**
20	ACL205C	**YPR194C (OPT2)**
21	ACR211W	**YDR380W (ARO10)**
22	ADL015C	**YNL104C (LEU4)**
23	ADL194W	**YOL141W (PPM2)**
24	ADL199W	**YML005W**
25	ADL258W	**YNL065W (AQR1)**
26	ADL328C	**YPR062W (FCY1)**
27	ADL362C	**YGR161C**
28	ADL392W	**YHR202W**
29	ADL397C	**YBR072W (HSP26)**
30	ADR020C	**YIL014W (MNT3)**
31	ADR079C	**YDL144C**
32	ADR119W	**YBR284W**
33	ADR142W	**YMR064W (AEP1)**
34	ADR199C	**YDR421W (ARO80)**
35	ADR205C	**YOR062C**

36	ADR229C	**YOR079C (ATX2)**
37	ADR237C	**YHL039W**
38	ADR286C	**YML004C (GLO1)**
39	ADR365W	**YOR337W (TEA1)**
40	AEL057C	**YCR023C**
41	AEL099W	**YDR338C**
42	AEL114C	**YJL163C**
43	AEL143W	**YCR028C (FEN2)**
44	AEL151C	**YJL190C (RPS22A)**
45	AEL171C	**YJL059W (YHC3)**
46	AEL345W	**YIL014W (MNT3)**
47	AER313C	**YDR452W (PPN1)**
48	AER338C	**YMR209C**
49	AER401W	**YDR368W (YPR1)**
50	AER409C	**YDR022C (CIS1)**
51	AER426C	**YIL137C**
52	AER444W	**YIL166C**
53	AER445C	**YJR076C (CDC11)**
54	AER459w	**YBR072W (HSP26)**
55	AFL158C	**YAL067C (SEO1)**
56	AFL160C	**YPL248C (GAL4)**
57	AFL204C	**YHR096C (HXT5)**
58	AFL235W	**YIL014W (MNT3)**
59	AFR045W	**YHR132C (ECM14)**
60	AFR206C	**YKL140W (TGL1)**
61	AFR228W	**YKR093W (PTR2)**
62	AFR322C	**YGR138C (TPO2)**
63	AFR333W	**YKL217W (JEN1)**
64	AFR415C	**YJR062C (NTA1)**
65	AFR437W	**YBR072W (HSP26)**
66	AFR506C	**YFL014W (HSP12)**
67	AFR548C	**YGL202W (ARO8)**
68	AFR561W	**YMR226C**
69	AFR633W	**YBR072W (HSP26)**
70	AFR675W	**YNL274C**
71	AFR726W	**YDR525W-A (SNA2)**
72	AGL027W	**YPR194C (OPT2)**
73	AGL049C	**YGR017W**
74	AGL050C	**YGR019W (UGA1)**

75	AGL087C	**YGL012W (ERG4)**
76	AGL142C	**YPL058C (PDR12)**
77	AGL143C	**YLR130C (ZRT2)**
78	AGL322W	**YBL039W-B**
79	AGL359C	**YER187W**
80	AGR108C	**YER176W (ECM32)**
81	AGR110W	**YER175C (TMT1)**
82	AGR235W	**YIL166C**
83	AGR250C	**YGR111W**
84	AGR297C	**YKR070W**
85	AGR329C	**YHR179W (OYE2)**
86	AGR407C	**YPL154C**

A. gossypii* genes absent from *S. cerevisiae* and *E. cymbalariae		
87	ABL004W	
88	ABL060C	
89	ABL062C	
90	ABL067C	
91	ABL157W	
92	ABL167C	
93	ABR042W	
94	ACL007C	
95	ACL049W	
96	ACL115W	
97	ACL133W	
98	ACL156W	
99	ACL204W	
100	ACR002C	
101	ACR141W	
102	ACR169C	
103	ADL011W	
104	ADL070C	
105	ADL112W	
106	ADL148C	
107	ADL359C	
108	ADL393W	
109	ADL400W	
110	ADR057W	
111	ADR100C	

112	ADR245W
113	ADR246C
114	ADR347C
115	AER099C
116	AER101W
117	AER160C
118	AER382W
119	AER455C
120	AFL013C
121	AFL028W
122	AFL126W
123	AFL137W
124	AFL164C
125	AFL233C
126	AFL234C
127	AFR048W
128	AFR070W
129	AFR079C
130	AFR164W
131	AFR170C
132	AFR288C
133	AFR311C
134	AFR476C
135	AFR622W
136	AFR740W
137	AFR742W
138	AFR746C
139	AGL037W
140	AGL048C
141	AGL084C
142	AGL146W
143	AGL277W
144	AGL309W
145	AGL366C
146	AGL367C
147	AGL368W
148	AGL369W
149	AGR013C
150	AGR055C

151	AGR090C
152	AGR124C
153	AGR404W
